# Disruption of c-di-GMP Signaling Networks Unlocks Cryptic Expression of Secondary Metabolites during Biofilm Growth in *Burkholderia pseudomallei*

**DOI:** 10.1128/aem.02431-21

**Published:** 2022-03-31

**Authors:** Grace I. Borlee, Mihnea R. Mangalea, Kevin H. Martin, Brooke A. Plumley, Samuel J. Golon, Bradley R. Borlee

**Affiliations:** a Department of Microbiology, Immunology, and Pathology, Colorado State University, Fort Collins, Colorado, USA; University of Nebraska—Lincoln

**Keywords:** diguanylate cyclase, c-di-GMP, biofilm, syrbactin, malleipeptin, *Burkholderia pseudomallei*

## Abstract

The regulation and production of secondary metabolites during biofilm growth of *Burkholderia* spp. is not well understood. To learn more about the crucial role and regulatory control of cryptic molecules produced during biofilm growth, we disrupted c-di-GMP signaling in Burkholderia pseudomallei, a soilborne bacterial saprophyte and the etiologic agent of melioidosis. Our approach to these studies combined transcriptional profiling with genetic deletions that targeted key c-di-GMP regulatory components to characterize responses to changes in temperature. Mutational analyses and conditional expression studies of c-di-GMP genes demonstrates their contribution to phenotypes such as biofilm formation, colony morphology, motility, and expression of secondary metabolite biosynthesis when grown as a biofilm at different temperatures. RNA-seq analysis was performed at various temperatures in a ΔII2523 mutant background that is responsive to temperature alterations resulting in hypobiofilm- and hyperbiofilm-forming phenotypes. Differential regulation of genes was observed for polysaccharide biosynthesis, secretion systems, and nonribosomal peptide and polyketide synthase (NRPS/PKS) clusters in response to temperature changes. Deletion mutations of biosynthetic gene clusters (BGCs) 2, 11, 14 (syrbactin), and 15 (malleipeptin) in parental and ΔII2523 backgrounds also reveal the contribution of these BGCs to biofilm formation and colony morphology in addition to inhibition of Bacillus subtilis and Rhizoctonia solani. Our findings suggest that II2523 impacts the regulation of genes that contribute to biofilm formation and competition. Characterization of cryptic BGCs under different environmental conditions will allow for a better understanding of the role of secondary metabolites in the context of biofilm formation and microbe-microbe interactions.

**IMPORTANCE** Burkholderia pseudomallei is a saprophytic bacterium residing in the environment that switches to a pathogenic lifestyle during infection of a wide range of hosts. The environmental cues that serve as the stimulus to trigger this change are largely unknown. However, it is well established that the cellular level of c-di-GMP, a secondary signal messenger, controls the switch from growth as planktonic cells to growth as a biofilm. Disrupting the signaling mediated by c-di-GMP allows for a better understanding of the regulation and the contribution of the surface associated and secreted molecules that contribute to the various lifestyles of this organism. The genome of B. pseudomallei also encodes cryptic biosynthetic gene clusters predicted to encode small molecules that potentially contribute to growth as a biofilm, adaptation, and interactions with other organisms. A better understanding of the regulation of these molecules is crucial to understanding how this versatile pathogen alters its lifestyle.

## INTRODUCTION

Burkholderia pseudomallei is a saprophytic bacterium that switches to a pathogenic lifestyle in a range of hosts causing melioidosis, an often-fatal disease that is common in Southeast Asia, Northern Australia, and other parts of the world (1, 2). Recent published estimates predict approximately 165,000 human cases of melioidosis annually with greater than 50% mortality in 79 countries where the pathogen is endemic (3). The environmental cues that serve as the impetus for B. pseudomallei to initiate this lifestyle change from saprophyte to pathogen in coordination with signaling cues are unknown, although, rain, humidity, and wind are thought be drivers of increased B. pseudomallei prevalence (4, 5). While these climatic factors are beginning to be defined in the context of the epidemiology of disease transmission, the cues and signal sensing systems in B. pseudomallei that contribute to this process are still largely unknown. Bacteria have evolved to sense and respond to their external environment and as a result have developed sophisticated signaling systems to rapidly adjust to their dynamic environment. One such elegant signaling cascade involves c-di-GMP, which has been shown to be an important secondary messenger in numerous bacterial pathogens (6–9).

To better understand c-di-GMP signaling in pathogenic *Burkholderia* spp. (10), we previously characterized 22 transposon insertional mutants predicted to be involved in B. pseudomallei 1026b c-di-GMP signaling (11). Two adjacent transposon mutants in a c-di-GMP phosphodiesterase (PDE), *cdpA* (I2284), and I2285, which is predicted to encode a protein with an HD-related output domain (HDOD) both exhibited a reduction in motility (11). Proteins containing HDOD domains are metal-dependent hydrolases that are distributed widely in bacteria and are often associated with proteins that have signaling and regulatory activity (12, 13). We also observed reduced biofilm formation at 30°C and increased biofilm formation at 37°C in a transposon insertion mutant in II0885, which encodes a predicted protein with diguanylate cyclase and phosphodiesterase (DGC/PDE) activity (11). The most significant phenotype with the greatest dynamic range in biofilm response was observed for a transposon insertional mutant of II2523, a predicted diguanylate cyclase (DGC). This mutant exhibited reduced biofilm formation at 30°C but paradoxically exhibited enhanced biofilm formation at 37°C (11). To further characterize these phenotypes in this study, we created in-frame deletions of *cdpA* (I2284, PDE), I2285 (HDOD protein), II0885 (hybrid DGC/PDE), and II2523 (DGC) to better understand how each of these genes contributes to c-di-GMP-regulated phenotypes such as biofilm formation and motility in B. pseudomallei. In some cases, we constructed site-directed mutations in these genes to identify specific amino acids that contribute to the phenotypes observed. In addition, we generated a series of mutant strains in the select agent excluded and attenuated strain Bp82 of B. pseudomallei. Bp82 is a Δ*purM* mutant of B. pseudomallei strain 1026b that is deficient in purine biosynthesis and is unable to replicate in human cells and has previously been shown to be fully attenuated in hypersusceptible animal models, which include Syrian hamsters and immune deficient mice (14, 15). The Bp82 deletion strains and their isogenic complements in addition to site-directed mutations in these genes provides a tool kit to safely study how these genes and changes in targeted amino acids contribute to c-di-GMP signaling, biofilm formation, and secondary metabolite production in a BSL2 lab. These strains also afford the opportunity to better understand the physiology of this bacterium regarding how it responds to temperatures that it would encounter growing as a saprophyte and during infection of a human host. By perturbing c-di-GMP signaling in mutant strains, we can study the biofilm matrix and surface-associated components that are differentially expressed in addition to cryptic metabolites that are not expressed in the parental bacterial cultures that are grown under standard laboratory conditions.

A secondary goal of this study was to enhance our understanding of c-di-GMP-mediated regulation under biofilm-inducing growth conditions. We performed RNA-seq and differential gene expression analysis of ΔII2523 in cells grown statically as biofilms at either 28 or 37°C. In addition to the studying genes that contribute to biofilm formation (e.g., polysaccharide biosynthetic gene clusters), this analysis also revealed a suite of genes that were differentially regulated that included several NRPS/PKS biosynthetic gene clusters (BGCs). Some of these clusters have been previously characterized; however, our mutational approach resulted in unlocking the expression of BGCs that have been previously described as cryptic with unknown functional roles. Recently, there has been a lot of interest in characterizing the metabolites produced by *Burkholderia* spp. and understanding their roles during growth, competition, survival, and infection of hosts (16–19). The BGC notation used here was originally described by Biggins et al. (16), and we have further characterized some of these BGCs in this research. To better evaluate the role of these cryptic BGCs, we generated combinatorial mutants of ΔII2523 with BGC cluster 2 (unknown nonribosomal peptide synthetase [NRPS]), cluster 11 (unknown NRPS), cluster 14 (syrbactin), and cluster 15 (malleipeptin) to evaluate the contribution of these BGCs to biofilm formation and the production of antimicrobial compounds. Overall, we sought to further delineate the complexity of c-di-GMP signaling and the potential contribution of cryptic secondary metabolism to various c-di-GMP-controlled phenotypes in B. pseudomallei.

## RESULTS

### Contribution of *B. pseudomallei* c-di-GMP genes to biofilm formation.

**FIG 1:**
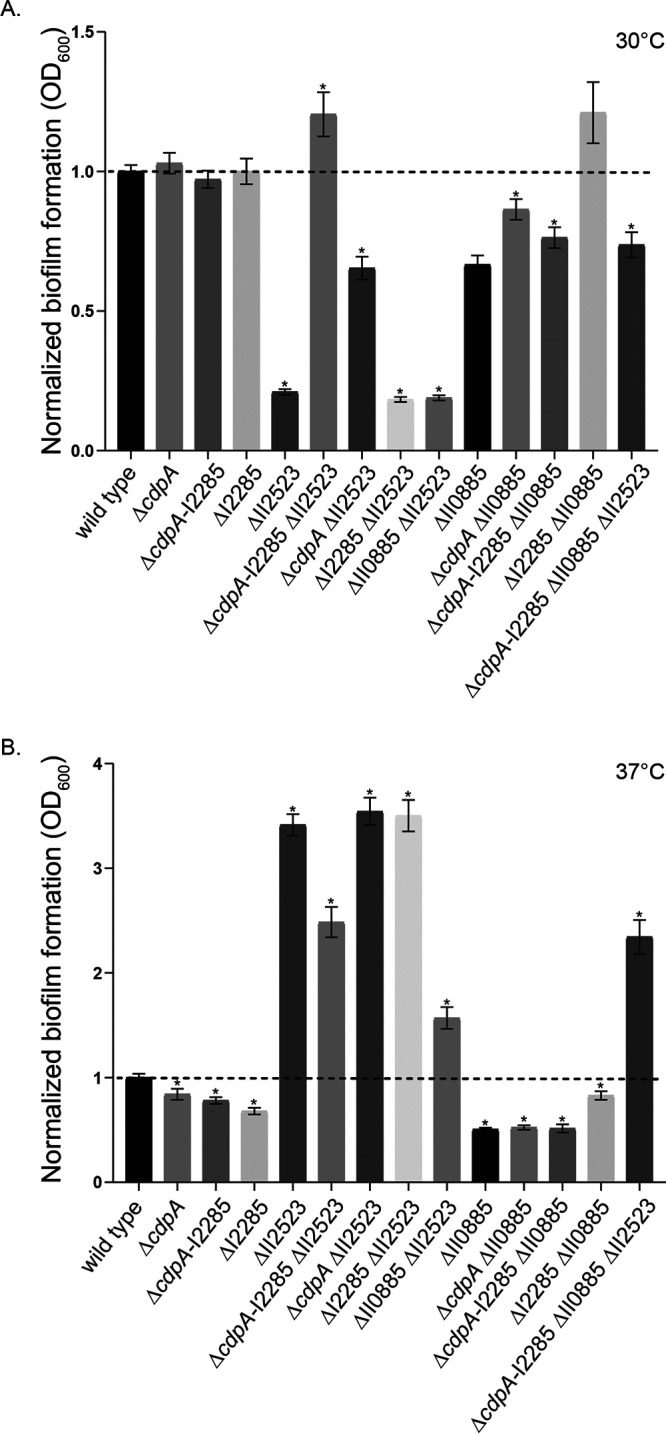
Biofilm formation of B. pseudomallei 1026b c-di-GMP deletion mutants. Wild-type and single, double, triple, and quadruple mutant strains were grown statically at 30°C (A) and 37°C (B) for 24 h. The data are representative of three independent experiments. Asterisks indicate a significance difference as determined with a Student *t* test utilizing the Bonferroni correction (*P* < 0.002) to account for multiple comparisons (*n* = 13).

**FIG 2:**
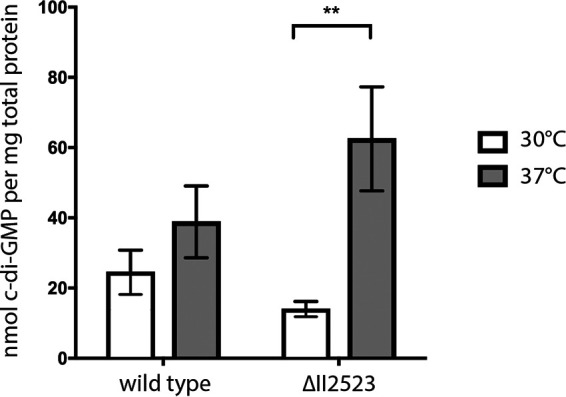
Quantification of c-di-GMP levels of B. pseudomallei 1026b and ΔII2523 grown statically at 30 and 37°C. *P* < 0.01. The statistical significance was determined using the Sidak-Bonferroni method across multiple Student *t* tests (**, *P* < 0.01). Error bars indicate the standard errors for three technical replicates. c-di-GMP extractions were repeated on separate days using two biological replicate cultures for each strain and temperature condition, with three technical replicates each.

In-frame deletion mutants of ΔII2523 were generated in the fully virulent wild-type B. pseudomallei 1026b and the attenuated Bp82 derivative to better understand the contribution of II2523 and additional c-di-GMP genes to biofilm formation. The ΔII2523 mutant recapitulated the temperature responsive biofilm phenotypes in both the B. pseudomallei 1026b (Fig. 1A), and the isogenic select agent excluded Δ*purM* strain B. pseudomallei Bp82 (see Fig. 10A and B). The data were comparable to the results that we had previously observed in a B. pseudomallei 1026b II2523 transposon mutant (11). The temperature-dependent biofilm formation phenotype of the ΔII2523 mutant could be in part attributed to c-di-GMP levels, which are elevated at 37°C and diminished at 30°C in comparison to the wild type (Fig. 2). Decreased biofilm phenotype of ΔII2523 at 30°C could be rescued with the Δ*cdpA* or with the Δ*cdpA*-I2285 (I2284-I2285) mutant but not with ΔI2285 alone, suggesting that the loss of the *cdpA* phosphodiesterase is sufficient to presumably elevate c-di-GMP levels in the ΔII2523 mutant (Fig. 1A). Loss of II0885, which is predicted to contain two MHYT, one EAL, and GGDEF domains and is most closely related to CdpA (38% identity at the amino acid level) was not able to rescue the ΔII2523 biofilm phenotype, suggesting that this protein does not work in cooperation either directly or indirectly with II2523 at 30°C (Fig. 1A). Interestingly, Δ*cdpA*, ΔI2285, and Δ*cdpA*-I2285 strains significantly reduced biofilm formation compared to the wild type, in addition to reducing biofilm formation in the ΔII2523 Δ*cdpA*-I2285 hyperbiofilm-forming background compared to ΔII2523 at 37°C (Fig. 1B). Neither Δ*cdpA* nor ΔI2285 mutants relieved the hyper biofilm of ΔII2523 at 37°C (Fig. 1B). Interestingly, ΔII0885 significantly reduced the ΔII2523 hyper biofilm formation phenotype resulting in levels that were more similar to wild-type levels suggesting that II0885 contributes to the hyperbiofilm-forming phenotype at 37°C (Fig. 1B). The deletion of II0885 had no effect in the quadruple mutant, Δ*cdpA*-I2285 ΔII0885 ΔII2523, which was identical to the triple mutant, Δ*cdpA*-I2285 ΔII2523, at 37°C (Fig. 1B); however, deletion of II0885 did decrease biofilm formation in all mutant combinations tested at 30°C (Fig. 1A).

### Contribution of *B. pseudomallei* c-di-GMP genes to motility.

**FIG 3:**
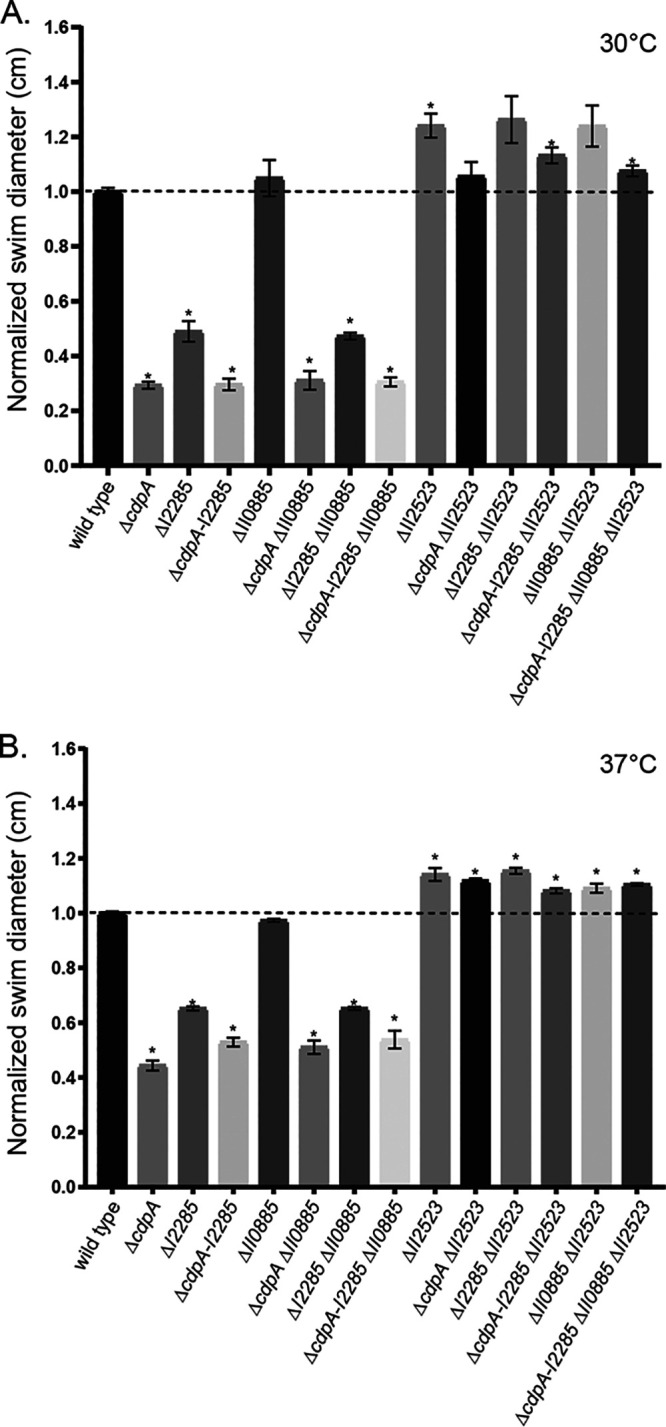
Swimming motility of B. pseudomallei c-di-GMP deletion mutants. Swimming motility of the wild type and single, double, triple, and quadruple mutant strains in 0.3% agar plates. The plates were incubated at 30°C (A) and 37°C (B) for 24 h. Asterisks indicate a significance difference determined using a Student *t* test with the Bonferroni correction (*P* < 0.002) to account for multiple comparisons (*n* = 13).

Differences in B. pseudomallei swimming were observed in plate-based motility assays. Δ*cdpA*, ΔI2285, Δ*cdpA*-I2285, Δ*cdpA* ΔII0885, and ΔI2285 ΔII0885 mutants and the triple mutant Δ*cdpA*-I2285 ΔII0885 all exhibited a decrease in swimming motility at both 30 and 37°C (Fig. 3). Decreased motility of strains with mutations in *cdpA* or mutations in orthologs of *cdpA* has been noted in several *Burkholderia* spp. (6, 11, 20, 21). Both ΔII2523 and the orthologous Δ*bcam2836* deletion in B. cenocepacia H111 exhibit increased motility (11, 21). Loss of II0885 did not alter the motility phenotypes of either the Δ*cdpA* or ΔI2285 single mutants, suggesting that *cdpA* and I2285 are epistatic to II0885 (Fig. 3). The loss of both *cdpA* and I2285 was not additive, suggesting that these proteins more than likely function in the same pathway (Fig. 3). Furthermore, *cdpA* and I2285 are not cotranscribed during planktonic growth, suggesting that these genes are regulated independently of each other under the conditions tested (see Fig. S1 in the supplemental material). The deletion of *cdpA* or I2285 did not significantly alter hypermotility in the ΔII2523 mutant background, suggesting that other phosphodiesterases or mechanisms also participate in the signaling that controls swimming motility (Fig. 3).

### Conditional expression of *cdpA* leads to decreased biofilm formation, whereas I2285 results in an increase in biofilm formation in *B. pseudomallei* Bp82.

**FIG 4:**
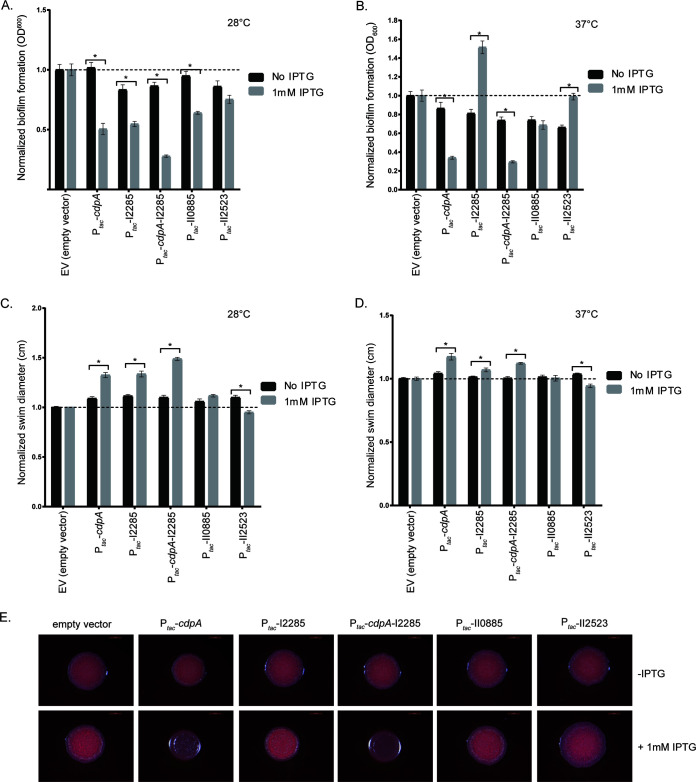
Biofilm formation, swimming motility, and colony morphology of B. pseudomallei Bp82 strains conditionally expressing *cdpA*, I2285, *cdpA* I2285, II0885, and II2523. Biofilm assays were incubated at 28°C (A) or 37°C (B) for 24 h. Swim assays were incubated at either 28°C (C) or 37°C (D) for 24 h. (E) Colony morphology on NAP-A plates incubated at 37°C. Images were taken after 3 days. Conditional expression of c-di-GMP genes was achieved by the addition of 1 mM IPTG.

To more rapidly evaluate the contribution of c-di-GMP genes to biofilm formation and the corresponding phenotypes, we constructed our mutants in B. pseudomallei Bp82, which is a select-agent excluded avirulent derivative of B. pseudomallei 1026b that can be used for research in a BSL2 laboratory (15). Subsequent temperature variation experiments with B. pseudomallei Bp82 were conducted at 28 and 37°C to approximate the temperatures that this opportunistic pathogen would, respectively, encounter residing in the environment and also during infection of a human host. IPTG (isopropyl-β-d-thiogalactopyranoside)-induced expression of *cdpA*, I2285, *cdpA*-I2285, and II0885, but not II2523, resulted in decreased biofilm formation at 28°C, suggesting that *cdpA*, I2285, *cdpA-*I2285, and II0885 may function to inhibit biofilm formation at 28°C (Fig. 4A). Strikingly, inducible expression of I2285 at 37°C resulted in a significant increase in biofilm formation not observed at 28°C, suggesting multiple functions for I2285 that are temperature dependent (Fig. 4A and B). Both *cdpA* and *cdpA-*I2285 expression reduced biofilm formation in the parental background at 37°C (Fig. 4B). Inducible expression of *cdpA* or *cdpA*-I2285 resulted in decreased biofilm formation at 28°C (Fig. 4A). Conditional expression of II0885 resulted in decreased biofilm formation at 28 and 37°C compared to the parental strain, while induction of II2523 enhanced biofilm formation compared to the uninduced control at 37°C (Fig. 4B). Conditional expression of either *cdpA*, I2285, or both *cdpA*-I2285 resulted in increased swimming diameter at 28 and 37°C (Fig. 4C and D), suggesting that these genes contribute to the regulation of swimming motility (Fig. 4C and D). Differences in swim motility were more noticeable at 28°C as opposed to 37°C (Fig. 4C). This is consistent with *cdpA* encoding a phosphodiesterase. Conditional expression of II2523, which encodes a putative diguanylate cyclase, resulted in decreased motility at both temperatures, whereas the conditional expression of II0885 had no effect (Fig. 4C).

Colony morphology was assessed to characterize phenotypes that are controlled by c-di-GMP (e.g., biofilm-forming capacity and exopolysaccharide production); however, in this study we did not observe striking differences in colony morphology for the c-di-GMP deletion strains grown on either LB, NAP-A, or YEM at either 28 or 37°C (see Fig. S2A to F). We also evaluated conditional expression as a means to evaluate the potential function of these genes. B. pseudomallei Bp82 strains with inducible expression of *cdpA*, I2285, *cdpA*-I2285, II0885, and II2523 were grown on LB, YEM, or NAP-A with agar and incubated at 28 and 37°C. There were no discernible differences in colony morphology on LB or YEM at either temperature or on NAP-A at 28°C (see Fig. S4A to C). However, IPTG-inducible expression of *cdpA* or *cdpA*-I2285 resulted in the notable loss of rugosity on NAP-A at 37°C, suggesting that *cdpA* can alter colony morphology (Fig. 4E).

We also evaluated the activity of these genes using a heterologous approach to evaluate the effect on c-di-GMP signaling-mediated phenotypes in Pseudomonas aeruginosa PAO1 (22–24). Inducible expression of *cdpA* or *cdpA*-I2285 in either P. aeruginosa PAO1 (see Fig. S3A) or the isogenic hyperbiofilm-former PAO1 Δ*wspF* resulted in decreased biofilm formation (see Fig. S3B). However, motility as measured by swim diameter was not significantly affected by conditional and heterologous expression in P. aeruginosa PAO1 (see Fig. S3C).

### Mapping residues important for activity in CdpA, I2285, and II2523.

**FIG 5:**
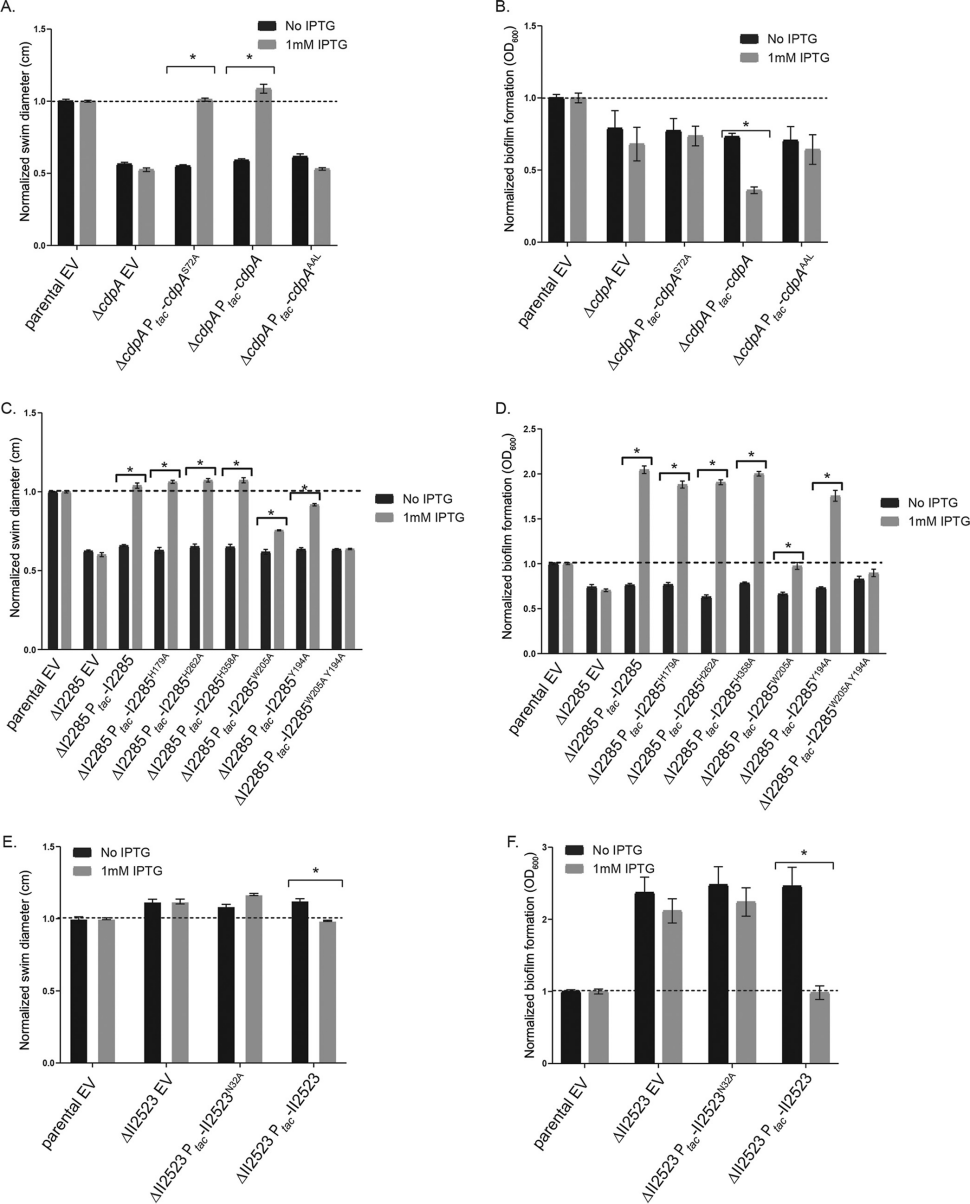
Site-directed mutations in *cdpA*, I2285, and II2523 identify amino acids that are important for swim and biofilm phenotypes in Bp82. (A and B) Swim (A) and biofilm (B) phenotypes of CdpA PAS4 (S72A) and EAL mutants. (C and D) Swim (C) and biofilm (D) phenotypes of I2285 mutants. (D and E) Swim (E) and biofilm (F) phenotypes of II2523 PAS4 (N32A) mutant. We used 1 mM IPTG to induce conditional expression. All assays were done at 37°C.

CdpA (I2284) is a predicted EAL-GGDEF hybrid that retains the canonical EAL domain, but the canonical GGDEF domain is replaced with the ASDFK residues, suggesting that this protein most likely functions solely as a phosphodiesterase rather than as both a diguanylate cyclase and a phosphodiesterase (6, 11). A single point mutation in *cdpA* to alter the EAL domain to AAL amino acid motif was constructed to assess the necessity of the EAL domain. Complementation of Δ*cdpA* with the *cdpA*^AAL^ construct did not restore motility to parental levels, suggesting that the EAL is important for CdpA-mediated motility (Fig. 5A). Complementation of Δ*cdpA* with an inducible wild-type *cdpA* decreased biofilm formation, suggesting that additional phosphodiesterase activity was responsible for biofilm inhibition (Fig. 5B). In addition to the EAL domain, CdpA also contains a PAS domain (11). PAS domains are sensory domains that can perceive signaling cues such as light, FMN, FAD, heme, and others (25). Strikingly, both CdpA and II2523 have PAS domains identified using Pfam analysis (11). CdpA does not retain a highly conserved asparagine but instead has a serine, while II2523 retains the conserved asparagine (26). Mutating the serine at position 72 to an alanine in the recombinant CdpA did not alter biofilm and swim motility in the Δ*cdpA* mutant during complementation (Fig. 5A and B).

Adjacent to *cdpA* (I2284) lies the gene locus I2285, which encodes a HDOD protein that also shares similarity to HD-GYP proteins based on protein alignment. We hypothesized that one of the histidine residues, which has been shown to be important in metal binding in HD-GYP proteins, might also be important for I2285 activity (11). We targeted three histidines (H179, H262, and H358) in I2285 for mutagenesis by replacing the respective histidine with alanine. None of these histidines contributed to the swim or biofilm phenotypes of induced I2285 (Fig. 5C and D). Sequence alignment of I2285 with GsmR, an HDOD protein from X. campestris, revealed additional amino acid residues to target for mutagenesis (see Fig. S5). Proteins with the HD-related output domain (HDOD) are widespread and can coordinate the signaling that controls chemotaxis (27). Liu et al. have previously shown that mutating a conserved tryptophan in GsmR resulted in loss of complementation in a swim assay (28). Mutating both the highly conserved tryptophan (W205) and a nearby tyrosine (Y194) in I2285 to alanine partially disrupted the increased motility of recombinant I2285 expression in the ΔI2285 mutant (Fig. 5C). However, incorporating both mutations (Y194A and W205A) completely disrupted complementation in both swim and biofilm assays (Fig. 5C and D). Interestingly, the single mutation of W205A did partially reduce the hyperbiofilm formation of overexpression of recombinant I2285, whereas Y194A did not (Fig. 5D). Mutation of the asparagine (position 32) of the PAS domain (11) resulted in a minor increase in ΔII2523 swimming motility, although it was not significant (Fig. 5E). This same PAS mutation in II2523 did not complement the ΔII2523 biofilm phenotypes at 37°C back to parental levels, indicating the necessity of this asparagine, whereas full-length II2523 was able to fully complement the hyperbiofilm phenotype of II2523 at 37°C (Fig. 5F).

### Transcriptional analysis of ΔII2523 and parental biofilms at 28 and 37°C reveals a multitude of differential expression genes among pairwise comparisons.

**FIG 6:**
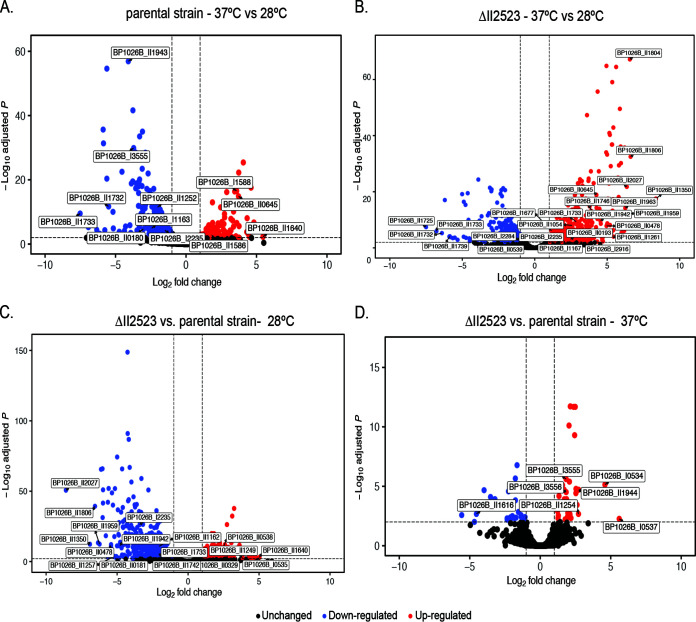
(A and B) Volcano plots of genes differentially regulated in the Bp82 parental strain (A) and Bp82 ΔII2523 (B) cells statically grown at 37°C versus 28°C. (C and D) Volcano plots of ΔII2523 at 37°C versus the parental strain at 37°C (C) and ΔII2523 at 28°C versus the parental strain at 28°C (D). Dashed lines represent cutoffs for a log_2_-fold change of <−1 or >1 (vertical) and an adjusted *P* value significance of <0.01 (horizontal).

Differential expression analysis of parental B. pseudomallei Bp82 biofilm formation at 37°C compared to 28°C exhibited numerous transcript changes after 24 h of static growth (see Table S2). We used the DESeq2 package (29) for RNA-seq differential expression analyses at log_2_-fold change thresholds of <−1 or >1 and an adjusted *P* value of <0.01. At these thresholds, 123 genes were significantly upregulated, with the highest being II1640 (T3SS-3 secretion system) at 44-fold, and 318 genes were significantly downregulated, with the lowest being II1733 (T3SS-2 secretion system) at −123-fold when comparing parental biofilm formation at 37°C versus 28°C (Fig. 6A; see also Table S2). Upregulated genes included pyochelin (II0645, cluster 10), malleipeptin (II1746, cluster 15), malleiobactin (I1735, cluster 1), *bec* biofilm cluster (I2923), and the T3SS-3, T6SS-3, and T6SS-6 secretion systems (Fig. 6A; see also Table S2). Genes downregulated included those involved in motility (I3555); T3SS-2; cluster 3 (I1164, unknown), cluster 7 (II0180, isonitrile), cluster 12 (II1252, bactobolin), and cluster 16 (II1943, unknown) NRPS/PKS biosynthesis; and a diguanylate cyclase I2235 (Fig. 6A; see also Table S2).

The greatest dynamic range in biofilm formation was observed in the ΔII2523 mutant when it was grown at temperatures that approximate human body temperature (37°C) compared to temperatures B. pseudomallei encounters growing as saprophyte (28 or 30°C) (Fig. 1A and B; see also Fig. 10A and B). Differential expression analyses of all B. pseudomallei transcripts indicated there were 443 genes that were upregulated with the highest being II1350 (syrbactin) at 328-fold and 312 genes that were downregulated, with the lowest being II1725 (T3SS-2) at −181-fold in this comparison (see Table S2). Genes that were upregulated at 37°C compared to 28°C included those involved in the biosynthesis of NRPS cluster 1 (I1733, malleiobactin, siderophore), cluster 2 (I1677, unknown), cluster 3 (I1167, unknown), cluster 10 (II0645, pyochelin), cluster 13 (II1261, unknown), cluster 14 (II1350, syrbactin), cluster 15 (II1746, malleipeptin), and cluster 16 (II1942, unknown); *bec* biofilm exopolysaccharide cluster (I2916); T6SS-4 and T3SS-3, capsules II (II0478) and III (II1959); *bcaA* (autotransporter); and I2235, a diguanylate cyclase (Fig. 6B; see also Table S2). Genes that were downregulated included motility genes, 2-alkyl-4-quinolone (II0539, cluster 9); T3SS-2, T3SS-3, and T6SS-5 secretion system genes; and *cdpA* (I2284) (Fig. 6B; see also Table S2).

At 28°C, ΔII2523 forms considerably less biofilm than the parent strain (Fig. 1A) (11), a phenotype that would be predicted if II2523 functioned as a diguanylate cyclase. Interestingly, a considerable number of genes (239 significantly up- and 547 downregulated) were altered when comparing the ΔII2523 to the parent grown at 28°C (see Table S2). Genes that were upregulated in ΔII2523 compared to the parent strain included capsule IV (I0535), genes involved in motility, malleilactone (II0329, cluster 8), 2-alkyl-4-quinolone (II0538, cluster 9), bactobolin (II1249, cluster 12), T6SS-5, T3SS-3, and *N*-acylhomoserine lactone synthase (*bpsI2*), with the most upregulated gene being II1640 (part of the T3SS-3 secretion system) (Fig. 6C; see also Table S2). Genes that were downregulated included cluster 1 (I1733, malleobactin), cluster 3 (I1162), cluster 7 (II0181, isonitrile), cluster 13 (II1257), cluster 14 (II1350, syrbactin), cluster 15 (II1742, malleipeptin), and cluster 16 (II1942); T6SS-4, capsule II (II0478) and III (II1959, *bce-I*); *bce-II* (II1896); and I2235 (diguanylate cyclase), with the most downregulated gene being II2027 (ortho-halobenzoate 1,2-dioxygenase alpha-ISP protein; OhbB) (Fig. 6C; see also Table S2). A number of the genes in this data set have been previously shown to be differentially regulated by quorum-sensing, including clusters 3, 8, 9, 12, and 13, along with capsule III (*bce-I*) and *bce-II* (30).

Thirty-six genes were significantly upregulated when we compared ΔII2523 grown at 37°C to the parental biofilm at 37°C (see Table S2). These genes included capsule IV, bactobolin biosynthesis (cluster 12), cluster 16 (unknown NRPS), and motility, with the most upregulated gene being I0537 (part of capsule IV) at 48.6-fold (Fig. 6D). Twenty-six genes, mainly T3SS-3, were downregulated when we compared ΔII2523 versus parental biofilms at 37°C, with one of the most downregulated genes being II1616 (a component of the T3SS-3 secretion system) at −23.1-fold (Fig. 6D; see also Table S2). Although considerably fewer transcripts were differentially expressed when ΔII2523 grown at 37°C was compared to the parental strain at 37°C, it is evident that this gene likely contributes to key physiological aspects of B. pseudomallei at this host-associated temperature.

### Visualization and validation of temperature-dependent global expression trends reveals significant differentiation in key *B. pseudomallei* functional clusters.

**FIG 7:**
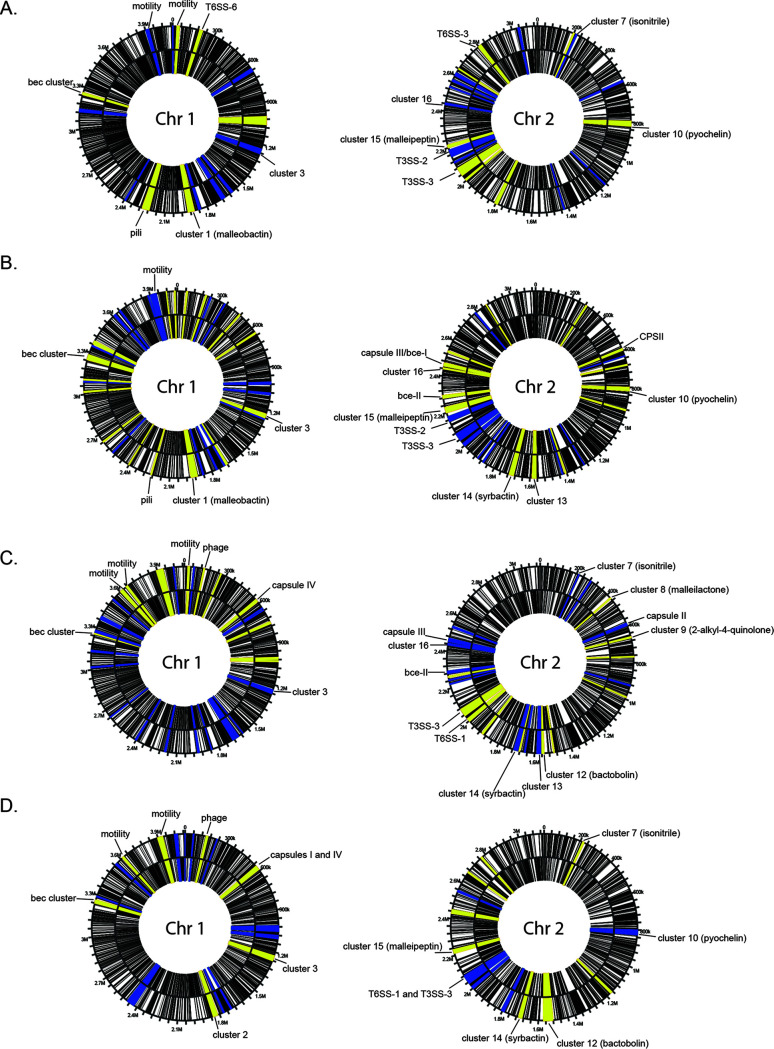
WoPPER analysis of gene clusters differentially regulated in the Bp82 parental strain and Bp82 ΔII2523 by chromosome. (A to D) Comparisons of the parental cells at 37°C versus 28°C (A), ΔII2523 at 37°C versus 28°C (B), ΔII2523 at 37°C versus the parental strain at 37°C (C), and ΔII2523 at 28°C versus the parental strain at 28°C (D). Yellow indicates gene clusters that were upregulated, and blue indicates gene clusters that were downregulated.

Using the Webserver for Position Related data analysis of gene Expression in Prokaryotes (WoPPER) (31) to visualize fold change data output from DESeq2, we identified differential regulation of NRPS clusters and biofilm-associated exopolysaccharide clusters across both B. pseudomallei chromosomes (Fig. 7). Cells of the parental strain grown as biofilms at 37°C compared to 28°C exhibited differential expression of motility-associated clusters, a pilus cluster, the *Burkholderia* exopolysaccharide cluster (bec), NRPS clusters 1 (malleiobactin) and 3, and T6SS-6 on chromosome I, whereas NRPS clusters 7 (isonitrile), 10 (pyochelin), 15 (malleipeptin), and 16, along with T3SS-2, T3SS-3, and T6SS-3, were differentially affected on chromosome II (Fig. 7A). A comparison of ΔII2523 at 37°C versus 28°C revealed some overlap of affected clusters on both chromosomes, and yet clusters 13 and 14 (syrbactin), as well as the biofilm-associated clusters CPSIII (*bce-I*), *bce-II*, and CPSII, represented features upregulated specifically in the ΔII2523 background (Fig. 7B). Further pairwise comparisons of ΔII2523 versus the parental strain at 37°C (Fig. 7C) and ΔII2523 versus the parental strain at 28°C (Fig. 7D) showed multiple NRPS, biofilm-associated, and capsule clusters differentially expressed especially on chromosome II, a trend we previously observed in B. pseudomallei supplemented with extracellular N-oxide signaling molecules (32). The graphical WoPPER analyses provide visual validation of RNA-seq data set pairwise comparisons, as well as additional orientation for the DESeq2 differential expression output (32).

Greater visual detail for these data sets and pairwise comparisons was achieved via heatmap analysis of complete predicted NRPS clusters (1 to 3, 5, and 7 to 16) and biofilm-associated clusters (CPSI, CPSII, CPSIII/*bce-I*, CPSIV, *bce-II*, and *becA-R*) (see Fig. S6). For the heatmap analyses, raw fold change values from DESeq2 were used as input for all transcripts spanning the predicted clusters (BGC cluster table), except for cluster 4, which is not present in B. pseudomallei 1026b, and cluster 6, which consists of four genes. The most striking patterns of differential regulation for complete clusters were evident for cluster 13, cluster 14 (syrbactin), CPSII, CPSIII/*bce-I*, and *bce-II* where ΔII2523 is similarly implicated in large negative shifts of expression especially at 28°C (see Fig. S6). Cluster 14 (syrbactin) was most strongly affected, at an average 82.4-fold upregulated for the entire cluster when comparing ΔII2523 at 37°C versus ΔII2523 at 28°C and −33.8-fold downregulated when comparing ΔII2523 at 28°C versus the parental strain at 28°C (see Fig. S6). Similarly strong trends were observed for the entire clusters CPSIII/*bce-I* and *bce-II*, which were upregulated at average fold changes of 47.2 and 35.9, respectively, when comparing ΔII2523 at 37°C versus ΔII2523 at 28°C, and downregulated at average fold changes of −43.2 and −22.1, respectively, when comparing ΔII2523 at 28°C versus the parental strain at 28°C (see Fig. S6).

**FIG 8:**
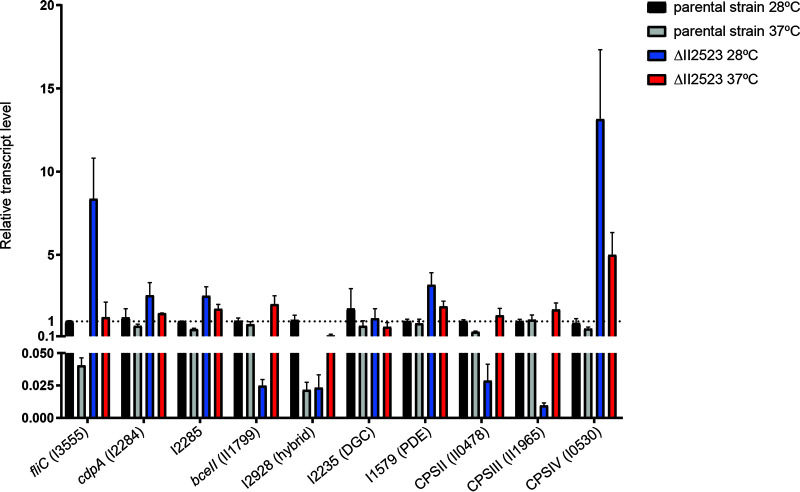
Gene expression (qRT-PCR) of *fliC* (I3555), *cdpA* (I2284), I2285, *bce-II*, (II1799), I2928 (hybrid), I2235 (DGC), I1579 (PDE), capsule II (II0478), capsule III (II1965), and capsule IV (I0530) of biofilm cells from ΔII2523 versus the parental strain grown at either 28 or 37°C. RNA samples for qRT-PCR assays were isolated after 24 h under conditions identical to those used for the RNA-seq data sets.

Representative transcripts from gene clusters were further validated via quantitative real-time PCR, which revealed similar expression trends compared to fold change differences between sample groups (Fig. 8). Although differential abundances calculated as a ratio of logs in pairwise comparisons and quantitative real-time PCR provides a value of one condition to the normal level, these complementary methods serve as further confirmation of RNA-seq analyses. Transcript levels relating to CPSII (II0478) and CPSIII (*bce-I*, II1965) were relatively reduced in ΔII2523 at 28°C by 0.029 and 0.010, respectively (Fig. 8). The capsule-associated loci, II0478 and II1965, were significantly downregulated in a similar fashion when comparing ΔII2523 to the parental strain at 28°C; −43-fold and −39-fold change reductions, respectively. II1799, part of the *bce-II* gene cluster, was decreased in ΔII2523 at 28°C at a relative transcript level of 0.025 compared to a relative transcript level of 2 for ΔII2523 grown at 37°C (Fig. 8). RNA-seq evaluation of II1799 revealed a −23.9-fold reduction in expression when comparing ΔII2523 to the parental strain at 28°C and a modest increase of 1.2-fold when comparing ΔII2523 to the parental strain at 37°C. *fliC* (I3555) expression for the parental strain grown at 37°C (Fig. 8) was reduced 0.04 relative to the parental strain, which agrees with the RNA-seq data set that indicated a −13-fold change for *fliC* at 37°C compared to 28°C. Capsule IV (I0530) expression was elevated in the ΔII2523 mutant grown at 28°C and less so at 37°C (Fig. 8). Correspondingly, I0530 was upregulated 18- and 4-fold when comparing ΔII2523 to the parental strain at 28 and 37°C, respectively, in the RNA-seq data sets (Fig. 8). Transcript levels for the EAL/GGDEF hybrid protein-encoding locus, I2928, adjacent to the *bec* biofilm-associated gene cluster was downregulated in the parental strain grown at 37°C (0.022 relative transcript) and ΔII2523 grown at 28°C (0.023 relative transcript) (Fig. 8). The relative transcript abundance for another c-di-GMP gene, I2284 (*cdpA*), was up 2.6-fold in the ΔII2523 mutant grown at 28°C. Thus, the relative transcript abundances for loci associated with biofilm formation follow a similar trend to the fold change expression differences observed in the comparative RNA-seq data sets across multiple conditions.

### Differential expression of diguanylate cyclase I2235 at various temperatures reflects a network of c-di-GMP regulation by II2523.

Four genes previously predicted to be involved in c-di-GMP signaling in B. pseudomallei (11) were differentially expressed, passing significance thresholds in our analyses (log_2_-fold change of <–1 or >1 and an adjusted *P* value of <0.01) (see Table S2). The phosphodiesterase *cdpA* was downregulated −2.2-fold in conditions of elevated c-di-GMP production (ΔII2523 at 37°C) compared to low c-di-GMP conditions (ΔII2523 at 28°C). I2235, a predicted diguanylate cyclase, was upregulated 3.8-fold in the same pairwise comparison (ΔII2523 at 37°C versus ΔII2523 at 28°C) (see Table S2). In comparing the parental Bp82 grown at 37°C versus 28°C, only the diguanylate cyclase I2235 was downregulated −2.8-fold (see Table S2). This diguanylate cyclase, I2235, was also downregulated −10.6-fold when we compared ΔII2523 to the parental strain grown at 28°C, while both *cdpA* (I2284) and the adjacent HD-like gene, I2285, as well as I3233, which encodes an ortholog of the flagellar brake protein, YcgR, were both upregulated 3.6- and 2.6-fold, respectively (see Table S2). Interestingly, none of the c-di-GMP genes were differentially expressed at our significance threshold when comparing ΔII2523 versus the parental strain at 37°C (see Table S2).

### Deletions in BGCs alter colony morphology and biofilm formation in the parental strain and ΔII2523.

**FIG 9:**
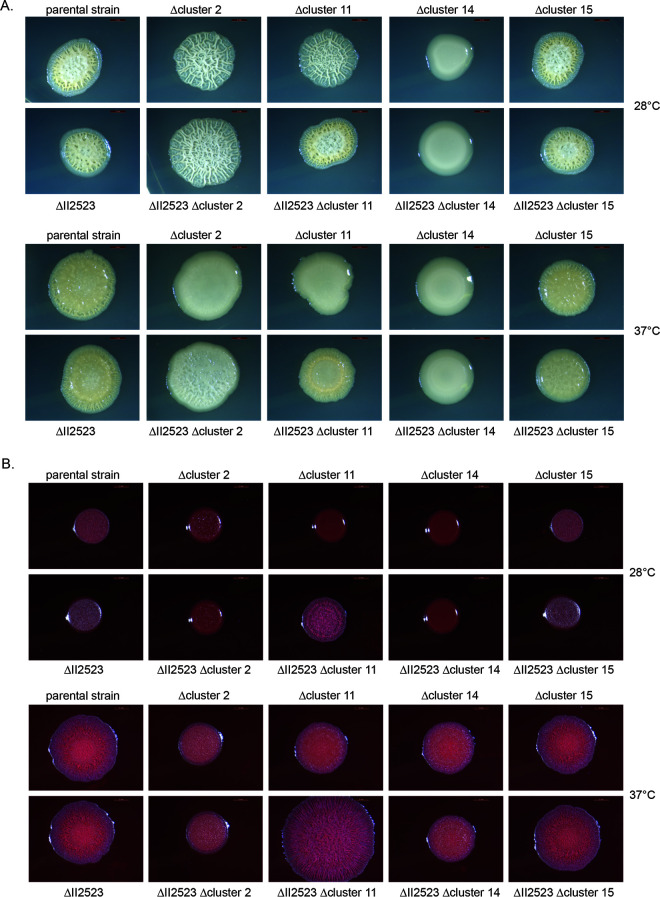
Colony morphology of NRPS/PKS mutants in the Bp82 parental strain or Bp82 ΔII2523 backgrounds on different media. Spots were grown on YEM (A) or NAP-A (B) at 28 or 37°C. Images were taken after 4 days of growth. Scale bar, 2 mm.

RNA-seq revealed differential regulation of numerous BGCs, suggesting that many of these may only be produced during growth as a biofilm and could also be regulated by c-di-GMP (see Table S3 for a description of BGCs). Four of these BGCs (clusters 2, 11, 14, and 15) have been characterized to various degrees in B. pseudomallei (16). Cluster 2 (I1663-1681) is predicted to encode a five-amino-acid lipopeptide that has yet to be characterized in detail (16) but was upregulated in only ΔII2523 at 37°C versus 28°C and versus the parental strain at 37°C (see Fig. S6). Cluster 11 (II1089-1108) encodes an unknown and predicted NRPS/PKS BGC, which was downregulated in both the parental strain and ΔII2523 at 37°C versus 28°C (see Fig. S6). Cluster 14 (II1345-1353) encodes the production of syrbactin, a known eukaryotic proteasome inhibitor, which was downregulated when comparing ΔII2523 at 28°C versus the parental strain at 28°C but was upregulated in ΔII2523 at 37°C versus ΔII2523 at 28°C, as well as in the ΔII2523 strain at 37°C versus the parental strain at 37°C (see Fig. S6). Cluster 15 (II1742-II1746) encodes malleipeptin, a lipopeptide and a potential biosurfactant (16). Genes for malleipeptin biosynthesis were upregulated for the parental strain and ΔII2523 at 37°C versus 28°C but downregulated when comparing ΔII2523 and the parental strain at 28°C (see Fig. S6). Together, these data suggest that BGCs contribute to B. pseudomallei biofilm formation; however, the exact mechanism for how these secondary metabolites contribute to biofilm formation has yet to be elucidated. We generated a series of deletion mutants of clusters 2, 11, 14, and 15 in both parental strain and ΔII2523 genetic backgrounds to evaluate the contributions of these secondary metabolites to biofilm formation. The loss of cluster 14 (syrbactin) in either the parental strain or the ΔII2523 background resulted in smooth colony morphology on NAP-A, YEM, or LB plates regardless of temperature, with the notable exception of NAP-A at 37°C (Fig. 9; see also Fig. S7). Most of the strains were rugose (wrinkly) on YEM agar at 28°C, with the exception of Δcluster 14 mutants (Fig. 9A). Cluster 2 deletion mutants were hyper-wrinkly at 28°C but the Δcluster 2 in the parental strain lost all rugosity at 37°C, while ΔII2523 Δcluster 2 retained some rugosity (Fig. 9A). When the medium conditions were altered, additional strains (Δcluster 2, ΔII2523 Δcluster 2, Δcluster 11, Δcluster 14, and ΔII2523 Δcluster 14) exhibited smooth colony morphology and increased pigmentation at 28°C as opposed to 37°C on NAP-A (Fig. 9B). Interestingly, we noted that ΔII2523 Δcluster 11 reproducibly produced increased radial growth on NAP-A at 37°C compared to other mutants on the same media (Fig. 9B).

**FIG 10:**
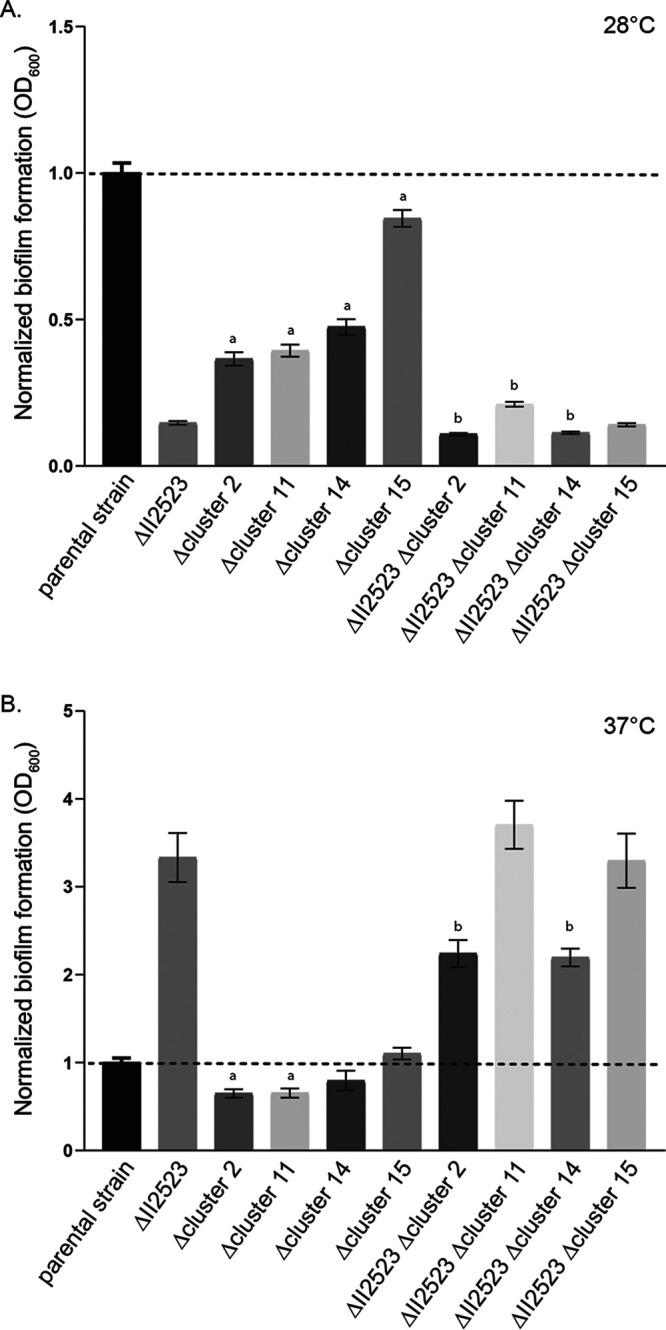
Biofilm formation of BGC Bp82 mutants either 28°C (A) or 37°C (B) for 24 h. Significance: a, statistical difference from the parental strain; b, statistical difference from ΔII2523.

As noted by Biggins et al., malleipeptin (cluster 15) is a biosurfactant and thus might play a role in biofilm formation (16). BGC deletion strains were evaluated for biofilm formation at both at 28 and 37°C (Fig. 10). Deletion of BGCs 2, 11, and 14 (syrbactin) and BGC 15 (malleipeptin) in the parental Bp82 background resulted in decreased biofilm formation, although to various degrees at 28°C, with the loss of cluster 15 exhibiting the smallest decrease (Fig. 10A). Loss of clusters 2 and 14 decreased biofilm formation in the ΔII2523 mutant background, while the loss of cluster 11 slightly enhanced biofilm formation in the ΔII2523 mutant at 28°C (Fig. 10A).

Only Δcluster 2 and Δcluster 11 exhibited less biofilm formation than the parental strain at 37°C (Fig. 10B). Double mutants ΔII2523 Δcluster 2 and ΔII2523 Δcluster 4 (syrbactin) exhibited decreased biofilm formation at 37°C (Fig. 10B) compared to ΔII2523. Notably, both ΔII2523 ΔBGC2 and ΔII2523 Δcluster 14 exhibited smooth colony morphology depending on the media and temperature (Fig. 9; see also Fig. S7).

Appropriately, two NRPS clusters (BGCs) encoding bactobolin (cluster 12) and an unknown metabolite (cluster 16) were upregulated in ΔII2523 compared to the parental strain at 28°C as observed in the RNA-seq data (Fig. 6D; see also Table S2). Bactobolin from B. thailandensis is a quorum-sensing mediated antibiotic effective against B. subtilis (33). Bactobolin from B. pseudomallei has been reported as a toxic polyketide-peptide hybrid molecule that interferes with host protein synthesis and is sensed by the bacterial feeding nematode, Caenorhabditis elegans (34). This potentially indicates that bactobolin could be an important molecule regulated by c-di-GMP that is secreted during biofilm formation to protect against predation and kill competing bacteria.

### BGCs contribute to growth inhibition of *B. subtilis* and *R. solani*.

**FIG 11:**
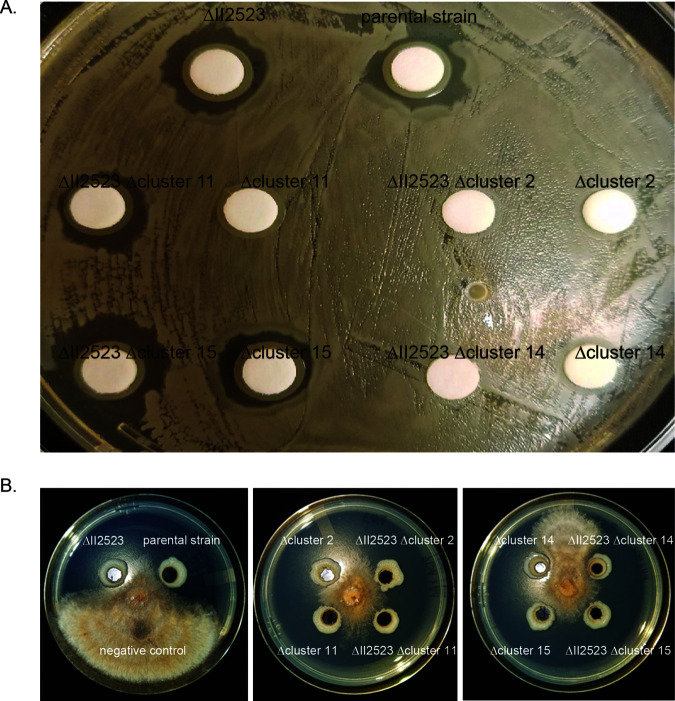
(A and B) Growth inhibition of B. subtilis (A) and R. solani (B). The zones of inhibition of B. subtilis and R. solani by the Bp82 parental strain or the Bp82 ΔII2523 NRPS deletion mutants are depicted. The B. subtilis images were taken after 24 h, whereas the R. solani images were taken after 5 days. All experiments were performed at 28°C.

Characterization of the biological activity of the secondary metabolites produced by BGCs 2, 11, 14 (syrbactin) and 15 (malleipeptin) from B. pseudomallei has been limited by the cryptic nature of the molecules encoded by these BGCs (16, 18). The deletion of BGCs 2, 11, 14, and 15 was assessed in competition with Bacillus subtilis and Rhizoctonia solani (a soilborne fungal plant pathogen) to evaluate growth inhibition. The parental strain, ΔII2523, Δcluster 15, and ΔII2523 Δcluster 15 equally inhibited growth of B. subtilis, suggesting that malleipeptin is dispensable (Fig. 11). Loss of cluster 2 or cluster 14 from either the parental strain or ΔII2523 backgrounds resulted in complete loss of B. subtilis growth inhibition, indicating that the cryptic secondary metabolite produced by cluster 2 and syrbactin (cluster 14) is important in inhibiting B. subtilis growth (Fig. 11). Interestingly, the loss of cluster 11 in the parental Bp82 background could not inhibit B. subtilis growth, while the ΔII2523 Δcluster 11 mutant was still able to produce metabolites that interfered with B. subtilis growth (Fig. 11).

The contribution of these B. pseudomallei secondary metabolites was also evaluated in the context of a eukaryotic organism, R. solani, a soilborne fungal pathogen of plants. All NRPS deletion mutants, except for the cluster 14 (syrbactin) deletion mutant, were able to strongly inhibit R. solani growth (Fig. 11). These data suggest that syrbactin plays an integral role in limiting the growth of a eukaryotic organism such as R. solani through an unknown mechanism. Purified syrbactin has been demonstrated to be a eukaryotic proteasome inhibitor in various eukaryotic cell lines (35); however, the mechanism of syrbactin inhibition of fungal growth is unknown. These data indicate that cluster 14 (syrbactin) is important for inhibition of the growth of B. subtilis and R. solani, while cluster 2 (uncharacterized NRPS) is important for specifically inhibiting B. subtilis, as shown in this study.

## DISCUSSION

B. pseudomallei is a versatile organism that can transition from a saprophytic lifestyle residing in the soil to a pathogen of human and animal hosts. This adaptable organism can also form robust biofilms and produce an array of secondary metabolites in response to various environmental parameters which include exogenous nitrate (32) and quorum-sensing signals (30). Correspondingly, the genome of B. pseudomallei encodes cryptic biosynthetic gene clusters (BGCs) that encode uncharacterized small molecules, although some metabolites have been characterized and produced using inducible promoters (16, 17). *Burkholderia* spp. have been reported to produce a diversity of metabolites that contribute to survival, adaptation, and interactions with other organisms (36). The vast majority of the environmental cues and signals that initiate the transition between lifestyles and regulate the production of metabolites are largely unknown in B. pseudomallei.

c-di-GMP has been shown to participate in controlling this switch in behavior or lifestyles at the level of a secondary messaging in many bacterial pathogens, including B. pseudomallei (6, 37–39). To better understand the role of c-di-GMP in biofilm dynamics and the regulation of secondary metabolites in B. pseudomallei, we followed up on our previous studies of bacterial responses to nitrate and temperature (11, 32, 40). In this study, we generated in-frame deletion mutants of a select group of c-di-GMP genes and conducted an epistatic genetic analysis in the attenuated strain of B. pseudomallei Bp82 to unmask phenotypes that might have been previously hidden. The aim of this study was to further characterize the role of specific c-di-GMP genes in motility, biofilm formation, and colony morphology by generating double, triple, and quadruple mutants in c-di-GMP metabolic genes, which has not been previously done in B. pseudomallei. Since the temperature-dependent biofilm phenotypes of the II2523 transposon mutant were recapitulated with in-frame deletion of II2523, we performed RNA-seq analysis to identify genes that are important for the transition of planktonic cells to a biofilm mode of growth in B. pseudomallei. The RNA-seq study revealed a suite of polysaccharides, BGCs, and secretion systems that mediate parental and ΔII2523 biofilm formation at 28 and 37°C.

The deletion of II2523 negatively affected biofilm formation at 28 and 30°C, resulting in decreased biofilm production as expected; however, this mutant hyperproduced biofilm at 37°C, as previously observed in our transposon mutational analysis (11). These biofilm and swim phenotypes also correlated with decreased levels of c-di-GMP at 30°C and correspondingly higher levels of c-di-GMP at 37°C. Future studies will continue to address this unknown mechanism now that we have demonstrated these phenotypes using in-frame deletion mutants that can be used in the BSL2 laboratory.

Much of what we have previously learned concerning function and regulation of these cryptic metabolites is gleaned from genomic comparisons of the foundational members of the Burkholderia pseudomallei complex (Bpc), which are comprised of B. pseudomallei, B. mallei, and B. thailandensis. Genomic analyses of Bpc organisms provide clues to the functional role of cryptic molecules given that these closely related species have divergent lifestyles and provide the opportunity to identify the key factors that contribute to survival in the different environments and hosts that they can inhabit. B. pseudomallei, which is the causative agent of melioidosis in humans, is an environmental saprophyte found in soils and surface waters in regions of endemicity (41). B. pseudomallei can also cause disease in a variety of mammalian hosts, birds, and reptiles (42) and can survive in amoebas (43). In contrast, B. mallei causes glanders in equids and is not found outside mammalian hosts, which is believed to be due to evolution through genome reduction and rearrangement from a single strain of B. pseudomallei (44). B. thailandensis is a soil-dwelling relative of B. pseudomallei that has been reported to cause infections in humans; however, it is generally considered to be less pathogenic, and reports of infections are rare (45–47). For this study, we focused on the B. pseudomallei 1026b genome, which is predicted to encode 15 NRPS/PKS BGCs, seven of which are not conserved in B. mallei (the genome-reduced relative of B. pseudomallei), and four of these BGCs are not conserved in B. thailandensis (16). The differences in the conservation of BGCs between these closely related species affords the opportunity to evaluate the contribution of these metabolites to survival in different niches (48).

In our RNA-seq data set under biofilm-inducing conditions, BGCs 2, 3, 12, and 13 were differentially regulated and were also and previously shown to be regulated by quorum sensing under planktonic growth conditions (30). However, very little is known about the functional role of these secondary metabolites in B. pseudomallei. In B. thailandensis, a malleilactone (BGC 8) mutant was significantly less virulent than wild-type B. thailandensis in a C. elegans killing model and did not inhibit D. discoideum from forming fruiting bodies (49). In B. pseudomallei K96243, a glidobactin (syrbactin) mutant was more susceptible to killing by human neutrophils. Interestingly, the glidobactin mutant was more lethal than the wild type at a high dose, while at a lower dose the survival between the mutant and wild type was comparable (18). This is in contrast to a report by Biggins and et al., who demonstrated that a syrbactin mutant in the B. pseudomallei 1026b background was highly attenuated in a mouse model (16). These contradictory results observed between the syrbactin mutants may be the result of the different genetic background of the strains used in these studies and/or the experimental parameters that were utilized.

The function of the various B. pseudomallei secondary metabolites encoded by the BGCs evaluated in these studies and their contribution to biofilm formation has yet to be fully elucidated. In this study, we have shown BGCs 2 and 11 make significant contributions to biofilm formation at 37°C. BGCs 2, 11, 14, and 15 also make significant contributions to biofilm formation at 28°C, where biofilm formation would provide a competitive advantage for survival in the environment. The Bpc group has recently undergone a proposed expansion to include *B. oklahomensis*, *B. humptydooensis*, *Burkholderia mayonis* sp. nov., and *Burkholderia savannae* sp. nov., in addition to B. pseudomallei, B. mallei, and B. thailandensis, based on whole-genome sequence analyses (44, 50). This expansion will increase our ability to further resolve the function and association of specific BGCs with organisms that inhabit different niches. These characterized BGCs could also provide better resolution for diagnostics based on genetic and metabolic markers.

The results of these studies illustrate the potential for how this system could be used to evaluate the secreted polysaccharides and secondary metabolites produced by B. pseudomallei, as observed from the effects mutations in key BGCs have on biofilm formation and colony morphology. Future studies will evaluate the role of these molecules in stabilizing and protecting the biofilm matrix from degradation. Other biofilm-producing strains, such as Pseudomonas aeruginosa, are known to have protein structural components that fortify the biofilm matrix (23) that are regulated by c-di-GMP and a protease inhibitor protein that protects the biofilm matrix from proteolytic attack (51). Biggins et al. proposed that malleipeptins (cluster 15) could function as biosurfactants/biofilm modulators, and the syrbactin-type proteasome inhibitors (cluster 14) were representative of small molecules that have gone unnoticed in B. pseudomallei. Based on the results of colony morphology and biofilm assays, it is clear that the molecules encoded by these BGCs alter the surfaces of these bacterial communities and manipulate biofilm dynamics.

Future studies will focus on the characterization of BGCs 12 and 16, since these clusters were upregulated in the ΔII2523 at 28°C and are likely important for bacterial competition and protection from predation during B. pseudomallei biofilm growth. The genetic backgrounds and environmental growth conditions identified in these studies will allow further chemical characterization of these cryptic metabolites and their role in growth, survival, competition, and infection of hosts.

## MATERIALS AND METHODS

### Bacteria growth, mutants, and complementation.

B. pseudomallei Bp82 (BSL3 select agent excluded strain) was grown in LB supplemented with 80 μg/mL adenine (LB+Ad80) (15). B. pseudomallei 1026b (select agent) was grown in LB and handled in a BSL3 laboratory. Generation of in-frame deletion mutants of Bp1026b_I2284, Bp1026b_I2285, Bp1026b_I2284-Bp1026b_I2285, Bp1026b_II0885, and Bp1026b_II2523 was accomplished by allelic exchange, as previously described (52). SOEing PCR was used to amplify ∼1 kB of flanking sequence on both sides of the gene(s) or a synthesized fragment for *bce-II* (GenScript). Deletion constructs for clusters 2, 11, 14, and 15 in pEXKm5 were kindly provided by D. DeShazer (16). The resulting fragments were cloned into pEXKm5 (52) and electroporated into RHO3. RHO3 pEXKm5 overnight cultures were grown in diaminophilic acid (DAP; 400 μg/mL) and kanamycin (35 μg/mL) and conjugated with Bp82. Merodiploids were selected on LB plus DAP plus 1,000 μg/mL kanamycin. Kanamycin-resistant clones were restruck onto plates containing LB, 1,000 μg/mL kanamycin, and 100 μg/mL 5-bromo-4-chloro-3-indolyl-β-d-glucuronide (X-Gluc) at 37°C. The following morning, several blue colonies were used to inoculate YT (8 g/L tryptone, 5 g/L yeast extract) broth containing 15% sucrose, grown with shaking for several hours at 37°C, and then plated onto YT (8 g/L tryptone, 5 g/L yeast extract, 15% sucrose) plates and 100 μg/mL X-Gluc. White colonies were restruck onto YT plates (15% sucrose, 100 μg/mL X-Gluc) at 37°C, and single colonies were patched onto LB plates with or without 1,000 μg/mL kanamycin. Kanamycin-sensitive clones with presumed deletions were verified using internal and external primers to a gene within the cluster or flanking the cluster of interest. For complementation and conditional IPTG expression studies, Bp1026b_I2284 (*cdpA*), Bp1026b_I2285, Bp1026b_I2284-Bp1026b_I2285, Bp1026b_II0885, and Bp1026b_II2523 were PCR amplified from Bp82 genomic DNA using Phusion DNA polymerase (NEB) or Kapa HiFi polymerase (Kapa Biosystems) using the primers (forward primers included a ribosome binding sequence) listed in Table S1 in the supplemental material. The corresponding fragments were ligated into the integration vector pUC18T-mini-Tn*7*T-km-LAC for expression in B. pseudomallei or P. aeruginosa (53). Clones were sequenced to confirm that no mutations had been introduced. The resulting vectors were electroporated into DH5α cells. Plasmids were isolated and electroporated in E. coli RHO3. RHO3 cells harboring the pUC18T-mini-Tn*7*T-KM-LAC constructs were grown in LB with 400 μg/mL DAP and 35 μg/mL kanamycin and mixed with RHO3 pTNS3 in Bp82 triparental matings. Complemented mutants and empty vector controls were selected on LB plates with 1,000 μg/mL kanamycin. A final 1 mM concentration of IPTG was used for gene expression. Site-directed mutagenesis was done according to the manufacturer’s recommendations using a QuikChange Lightning kit (Agilent Genomics), as previously described (11), using the oligonucleotides listed in Table S1. All plasmids were confirmed via sequencing.

### Static biofilm and swim motility assays.

Biofilm and swim motility assays were performed as previously described (11), with the addition of adenine to the media. P. aeruginosa strains (PAO1 or PAO1 Δ*wspF*) conditionally expressing Bp1026b_I2284 (*cdpA*), Bp1026b_I2285, Bp1026b_I2284-I2285, Bp1026b_II0885, Bp1026b_II2523, or empty vector (pUC18T-mini-Tn*7*T-KM-LAC) were grown overnight in LB (with kanamycin [300 μg/mL], where appropriate).

### c-di-GMP measurements.

c-di-GMP extractions and quantification were performed as described by Mangalea et al. (40), with modifications using the BSL-3 parental strain 1026b and an in-frame deletion mutant ΔII2523. Briefly, overnight cultures were grown in LB at 37°C with shaking at 250 rpm, diluted 1:50 in M9 salts minimal medium, and grown statically for 18 h at either 30 or 37°C. Extractions were performed using a chilled matrix buffer solution of acetonitrile/LC-MS methanol/LC-MS H_2_O/1% formic acid, supplemented with 100 nM 2-chloro-adenosine-5′-*O*-monophsphate (2-Cl-5′-AMP; Axxora, LLC) for internal standardization. Final absolute nucleotide concentrations were normalized to total protein concentrations using a Pierce 660-nm protein assay (Thermo Scientific). Extraction experiments were repeated on separate days using two biological replicate cultures for each strain and temperature condition, with three technical replicates each. The statistical significance was calculated using an unpaired Student *t* test in GraphPad Prism (v7) with the Bonferroni-Sidak correction for multiple comparisons.

### RNA isolation and RNA sequencing.

Total RNA was collected from pellicle biofilms formed at the air-liquid interface from three technical replicates per biological sample grown in six well Costar plates for 24 h at either 28 or 37°C. Samples in which pellicles were inhibited due to treatment were collected from bacterial cells at the bottoms and sides of the Costar plate. Each plate was seeded with three mutant replicates, and three parental strain replicates for each condition were tested. Then, 1.5-mL samples were collected, spun at 12,000 rpm for 2 min, and resuspended in 350 μL of RNAprotect bacterial reagent (Qiagen). Samples were kept on ice from this point forward. Samples were spun at 5,000 × *g* for 10 min, and the supernatant was discarded before resuspension in 1.5 mL of QIAzol reagent (Qiagen). Screw-cap tubes were prepared for each sample by adding ∼250 μL of sterile beads. The QIAzol suspension mixture was added, followed by incubation for 5 min at room temperature. Sample tubes were transferred back to ice before 3 rounds of 60-s bead beating on a TissueLyser2 (Qiagen). Samples were incubated at room temperature for 5 min before 200 μL of chloroform (Fisher Scientific) was added, and samples were then vortexed for 5 s before another room temperature incubation for 5 min. Samples were spun at a relative centrifugal force of 10,000 for 10 min; 500 μL of the aqueous phase was then removed and transferred to separate tubes containing 500 μL of molecular-grade 70% ethanol. RNA was subsequently extracted from the samples using the RNeasy kit (Qiagen), where the three technical replicates were pooled onto a single RNeasy column. Genomic DNA was removed using DNase I via two rounds of treatment using a Turbo DNA-free kit (Ambion). rRNA was removed using RiboZero rRNA removal reagents for Gram-negative bacteria (Illumina). Libraries were prepared following the RNA-seq sample work flow using the Scriptseq Complete kit for bacteria and indexed with unique Scriptseq Index PCR primers (Illumina). Samples were analyzed on a Tapestation using HS D1000 tapes and reagents (Agilent) to determine the average sizes and concentrations of the libraries. Size and molarity estimates were used to pool all libraries in equimolar concentrations. Final quality control and qPCR analyses were completed at the Colorado State University NGS Core Facility. A NextSeq run was completed on the pooled libraries using the NextSeq 500 Hi-Output v2 75-Cycle kit and Buffer Cartridge (Illumina). Sequence files were downloaded from the NGS server, demultiplexed according to index primers, and converted to FastQ files before initial quality control using FastQC (version 0.10.1) (54). Adapter sequences were trimmed using Trimmomatic (version 0.35) (55) before another quality control round using FastQC. Bowtie2 (56) was used to align sequencing reads to the reference genome GCF_000260515.1, and ASM26051v1 assembly (NCBI) and TopHat (57) were used for transcriptome mapping. HTseq (version 0.11.0) (58) was used to count accepted hits before the DEseq2 (version 1.20.0) (29) package was employed in R for comprehensive differential expression analysis. Raw read count coverage values were used to compare the differential gene expression between temperature treatments, mutants, and untreated controls. Using a negative binomial distribution to estimate variance and a Bayesian approach for variance shrinkage, the DEseq2 package produced logarithmic fold change values between the conditions tested. Wald tests were used to calculate the *P* value, and the Benjamini-Hochberg multiple testing correction was used to correct for the false discovery rate.

### Gene expression by quantitative real-time PCR.

RNA was isolated using identical conditions to the samples used for RNA sequencing. Briefly, genomic material was isolated from static cultures grown for 24 h at the temperature indicated using RNAprotect bacterial reagent (Qiagen) and QIAzol lysis reagent (Qiagen) before purification with RNeasy minikit columns (Qiagen). Total RNA samples were treated with DNase I (Ambion/Life Technologies) twice, and cDNA was synthesized using a Transcriptor first-strand cDNA synthesis kit (Roche) according to the protocol recommended by the manufacturer. Primers were designed using the PrimerQuest tool (Integrated DNA Technologies). The 23S rRNA subunit was used for normalization in all experiments, using previously published primers (59). qRT-PCR experiments were performed on a LightCycler 480 instrument (Roche) in 96-well plates, using technical triplicates, with the reference gene present on all plates. Three biological replicates were included in all analyses. As a control for genomic DNA contamination in the cDNA samples, control samples containing no reverse transcriptase enzyme were used in all experiments. Data were analyzed using the Pfaffl method (60), which considers the PCR efficiency percentage of all primer pairs, including the reference gene.

### Colony morphology.

Overnight cultures of B. pseudomallei strains were grown in LB. Then, 3-μL portions were spotted onto either LB, NAP-A (61), or YEM (62). The plates were incubated at either 28 or 37°C for 3 days. Colony morphology images were taken with a Leica MZ95 microscope.

### Growth inhibition assay of *Bacillus subtilis* and *Rhizoctonia solani*.

B. pseudomallei and B. subtilis 3610 overnight cultures were grown at 37°C with shaking (250 rpm). B. subtilis was diluted to a final optical density at 600 nm (OD_600_) of 0.1, and 100 μL of diluted culture was spread onto (LB+Ad80) plates until completely dry. Sterile filter discs (Remel, Lenexa, KS) were placed with sterile forceps onto plates, and 20-μL B. pseudomallei cultures, diluted to an OD_600_ of 0.5, were dispensed onto the filter discs. The plates were allowed to incubate at 28°C overnight. R. solani AG2-2 IIIB R-9 (J. Leach, Colorado State University) was propagated on potato dextrose agar (PDA) and grown at 28°C. Agar plugs were removed from the PDA+Ad80 and filled with 50-μL portions of overnight cultures of the parental strain or ΔII2523 NRPS mutants. Then, an agar plug of a 3- to 5-day-old culture of R. solani R-9 was placed in the middle of the plate. The plates were incubated at 28°C for 5 days.

### Data availability.

The transcriptomic data sets consisting of 12 raw Illumina sequence files associated with this study can be accessed at the GenBank Sequence Read Archive (SRA) (https://www.ncbi.nlm.nih.gov/sra) under accession number PRJEB47008.
