# Supplementary material for: Disruption of c-di-GMP Signaling Networks Unlocks Cryptic Expression of Secondary Metabolites during Biofilm Growth in Burkholderia pseudomallei
Source: Appl Environ Microbiol. 2022 Mar 31;88(8):e02431-21. doi: 10.1128/aem.02431-21 (PMC9040570; doi:10.1128/aem.02431-21)
Supplement: Supplemental file 1 — Fig. S1 to S7, Tables S1 and S3, and supplemental methods. Download aem.02431-21-s0002.pdf, PDF file, 9.1 MB [file aem.02431-21-s0002.pdf]

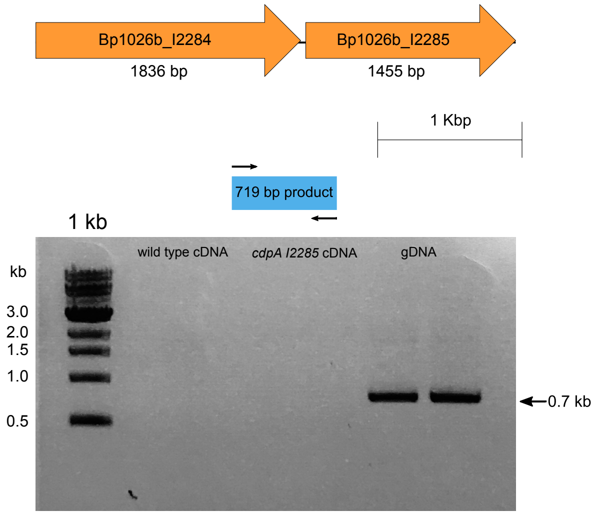

Fig S1 CdpA (I2284) and I2285 are not co-transcribed. cDNA from wild type, the Δ*cdpA*-I2285 double mutant, and wild-type genomic DNA were amplified to produce an expected product of 719bp. This product is only produced in genomic DNA where bands are visible for technical duplicate samples.

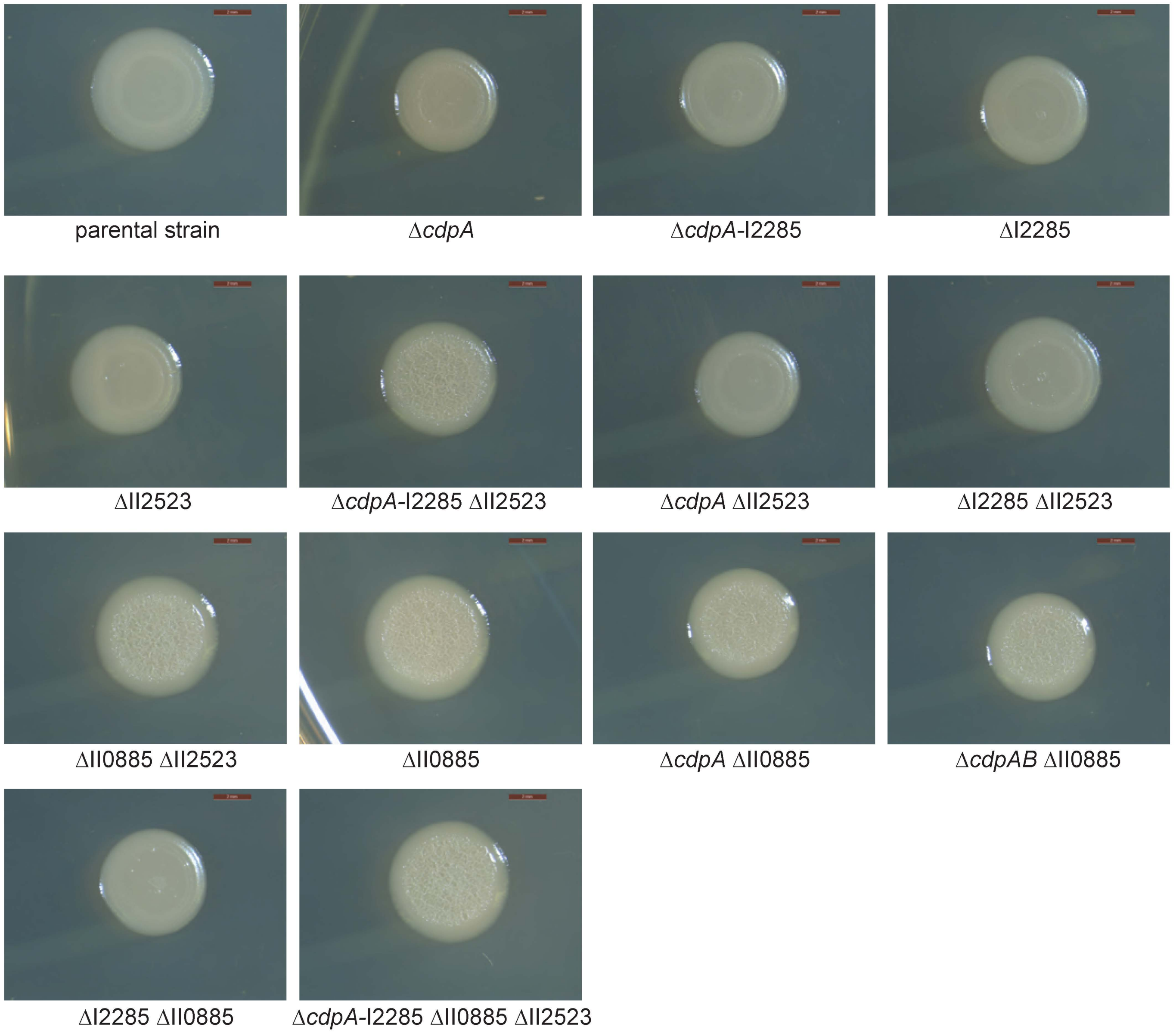

Fig S2A Colony morphology of c-di-GMP deletion mutants grown on LB at 28°C. Images were taken at day three.

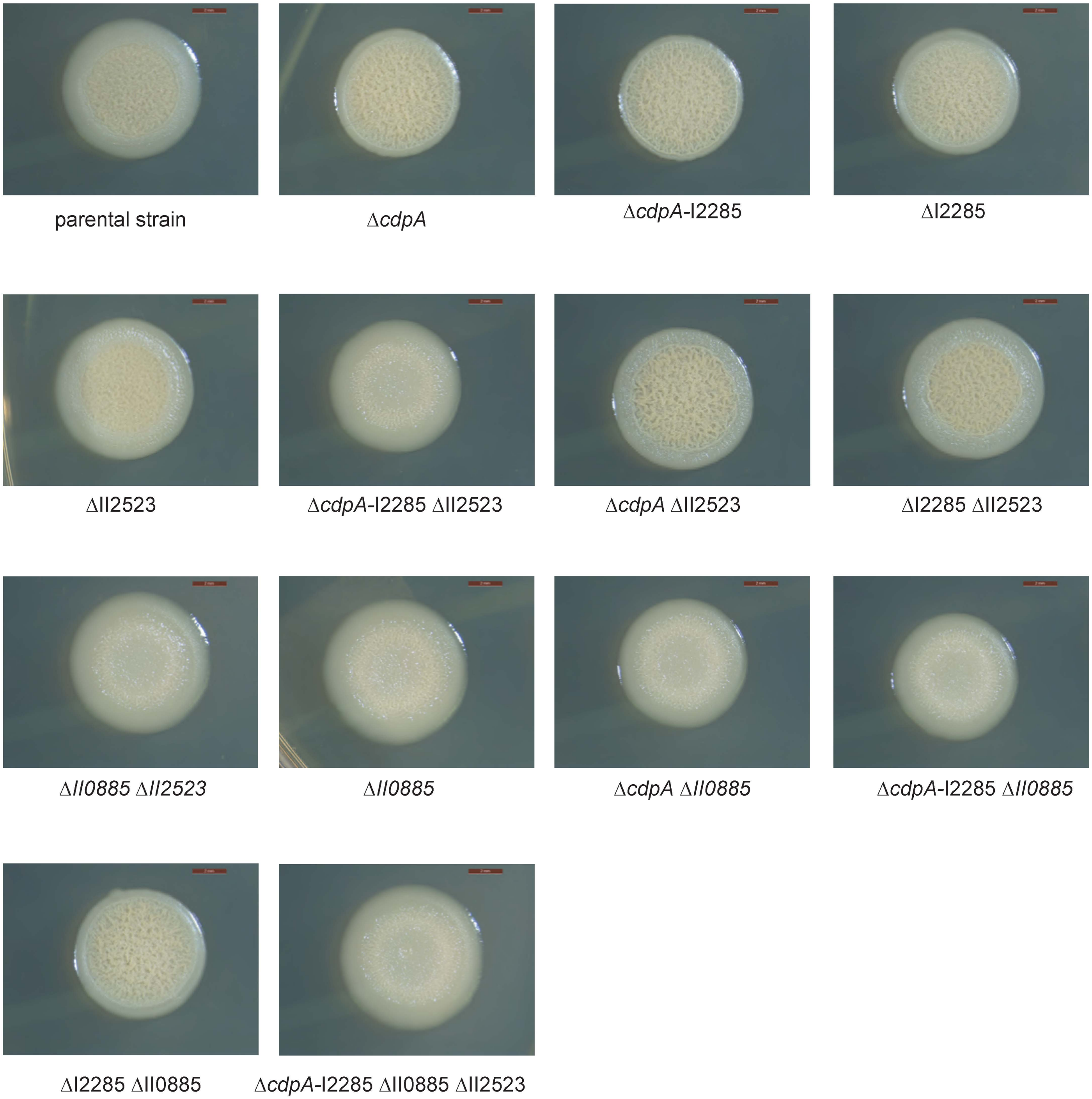

Fig S2B Colony morphology of c-di-GMP deletion mutants grown on LB at 37°C. Images were taken at day three.

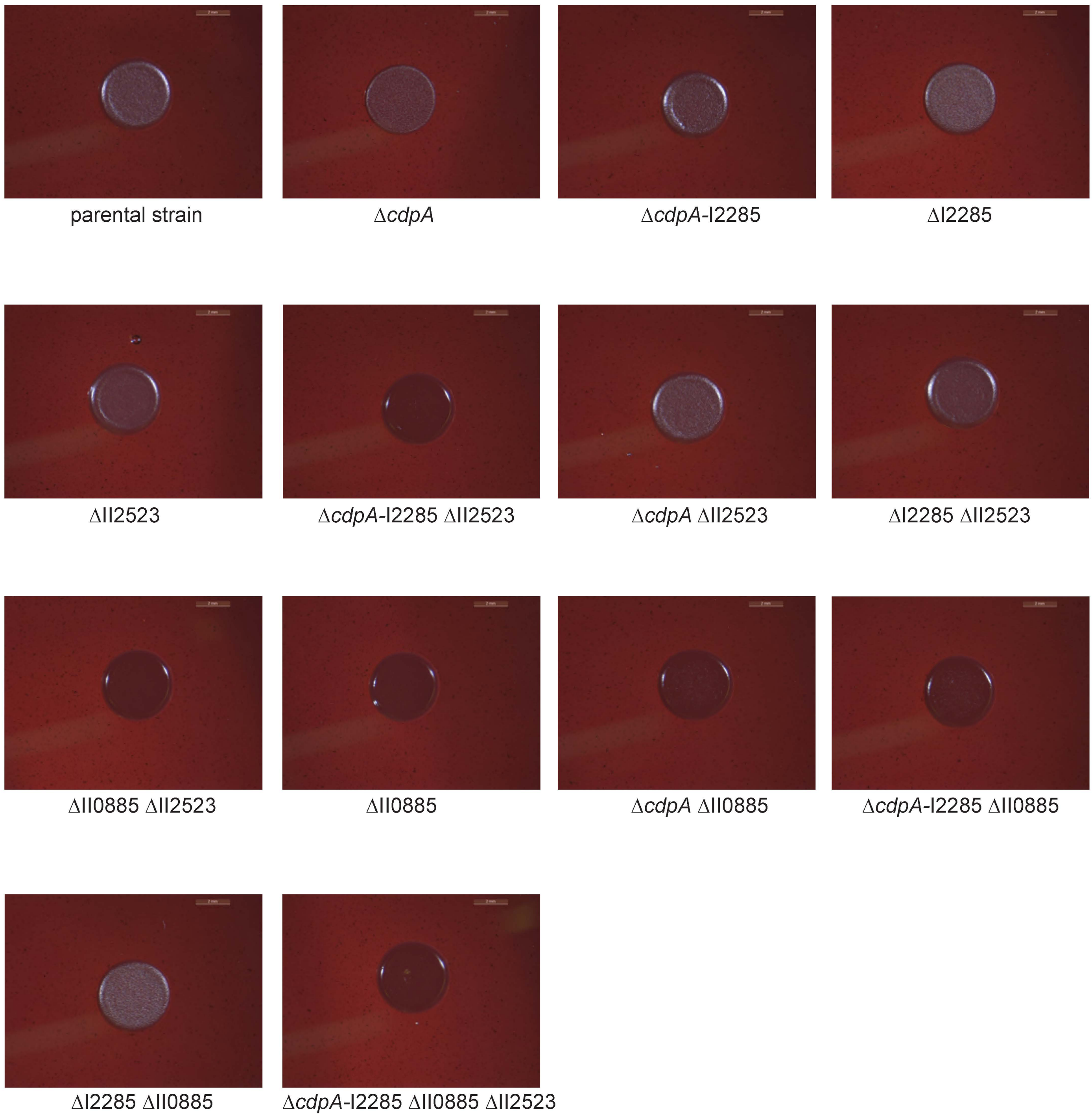

Fig S2C Colony morphology of c-di-GMP deletion mutants grown on NAP-A at 28°C. Images were taken at day three.

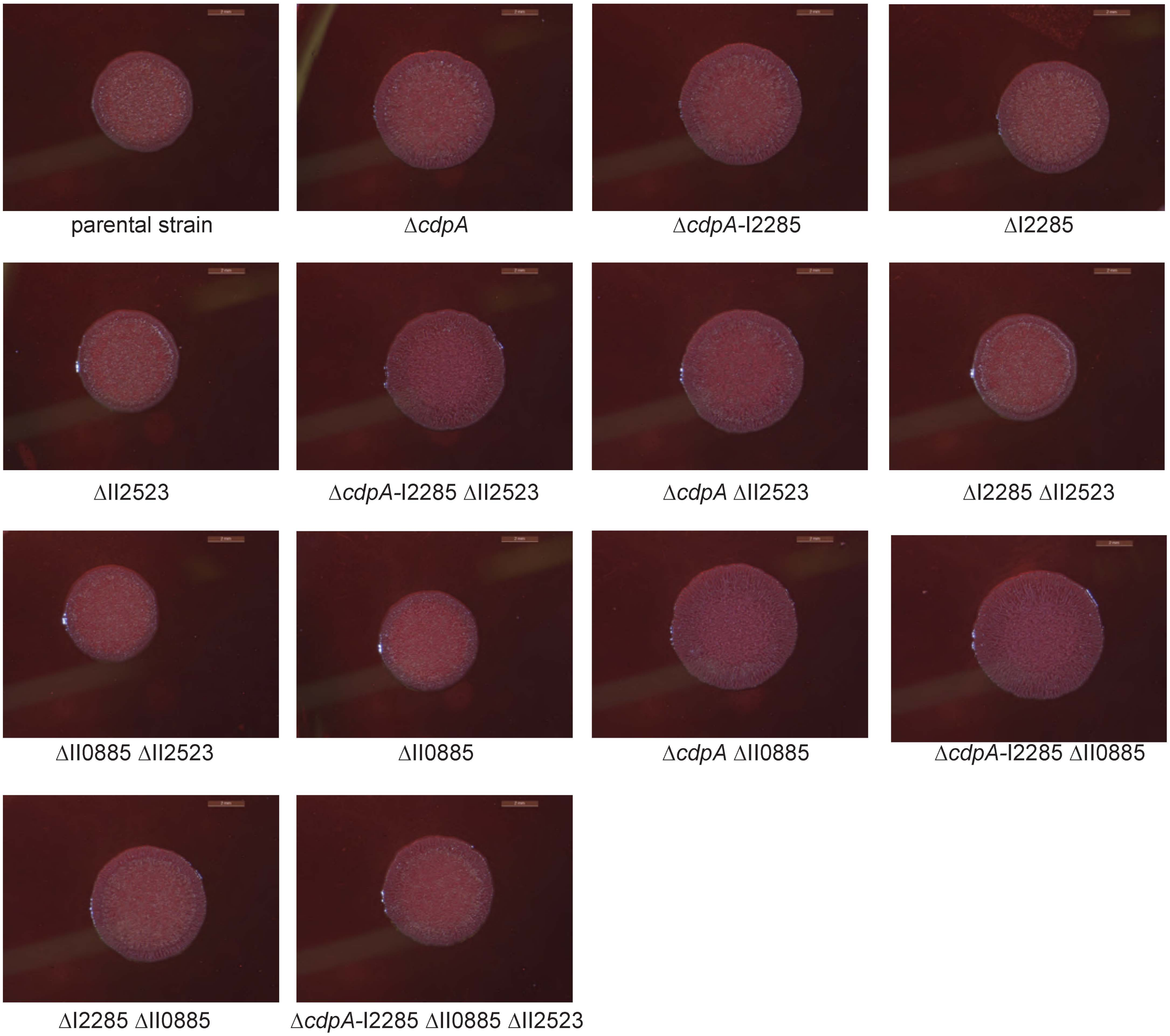

Fig S2D Colony morphology of c-di-GMP deletion mutants grown on NAP-A at 37°C. Images were taken at day three.

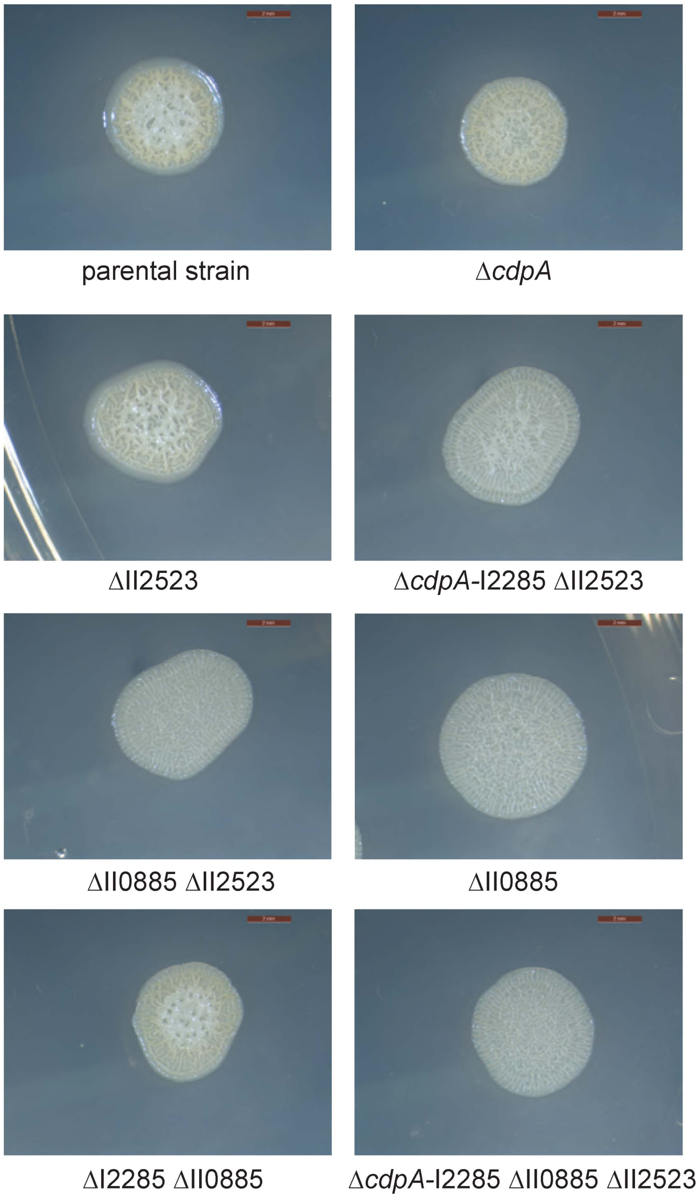

Fig S2E Colony morphology of c-di-GMP deletion mutants grown on YEM at 28°C. Images were taken at day three.

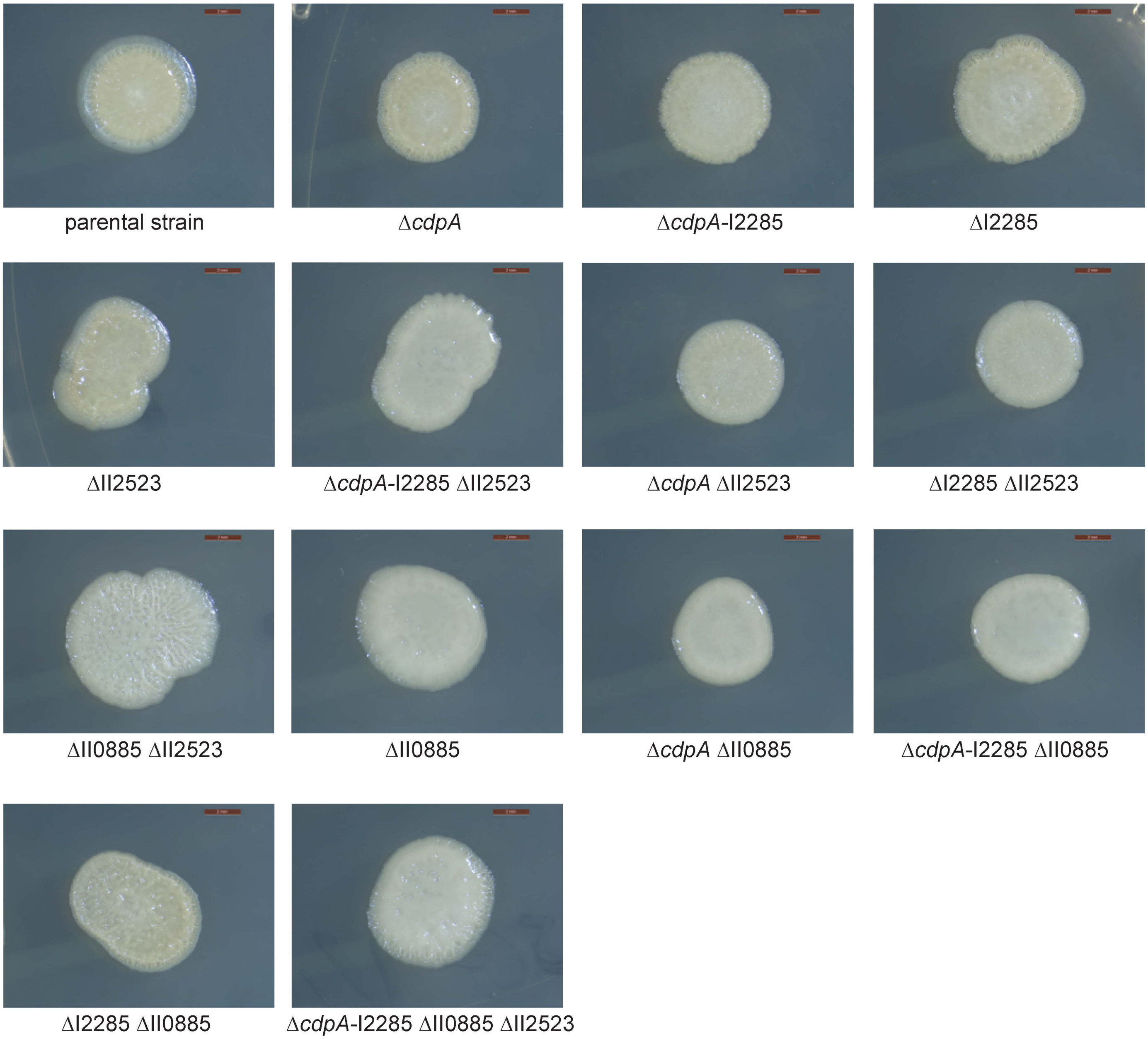

Fig S2F Colony morphology of c-di-GMP deletion mutants grown on YEM at 37°C. Images were taken at day three.

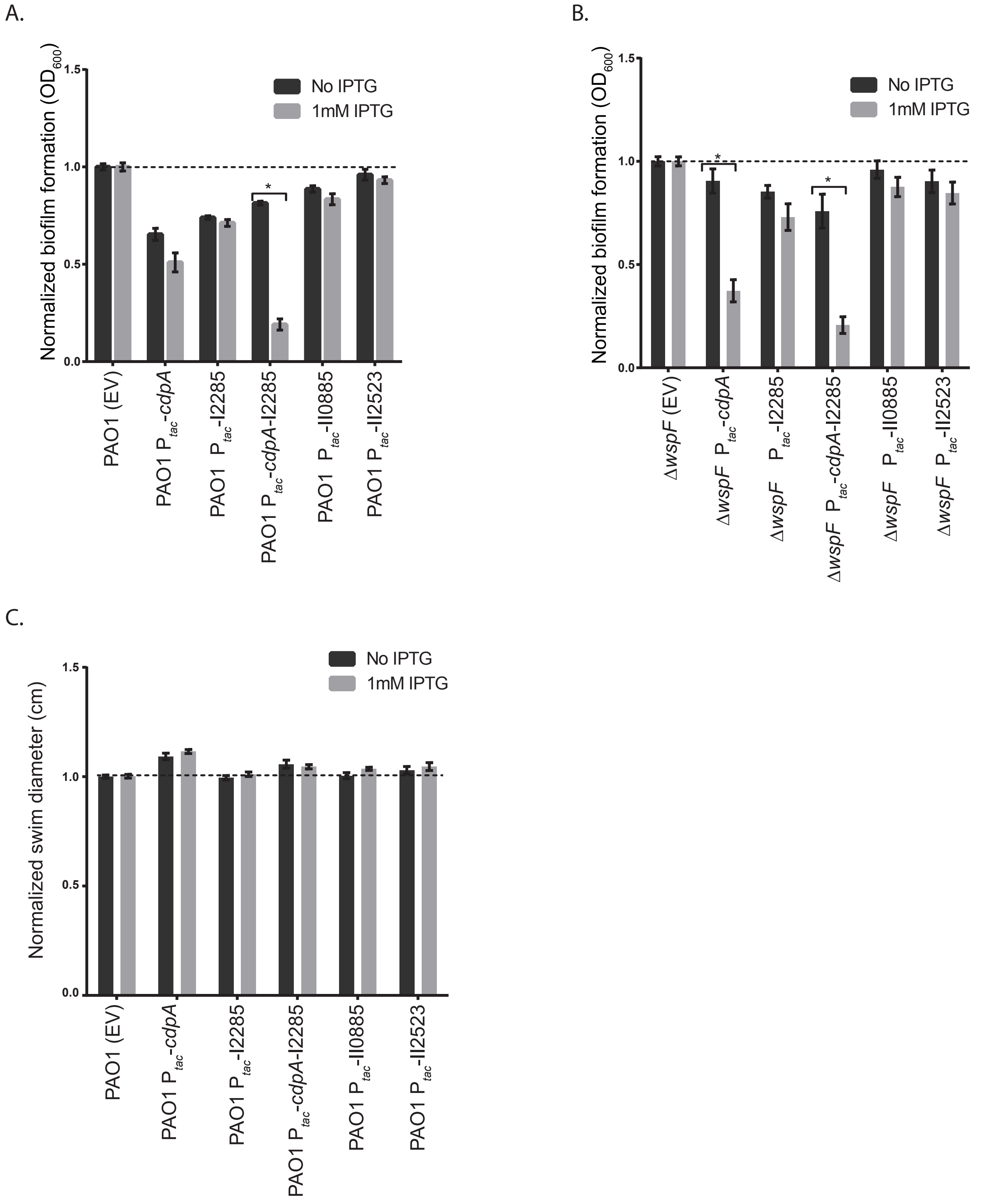

Fig S3 Biofilm formation and swimming motility of PAO1 and PAO1 Δ*wspF* strains heterologously expressing *cdpA*, I2285, *cdpA*-I2285, II2523, and II0885. Biofilm formation PAO1 (A) and PAO1 Δ*wspF* strains (B). Swimming motility of PAO1 strains (C).

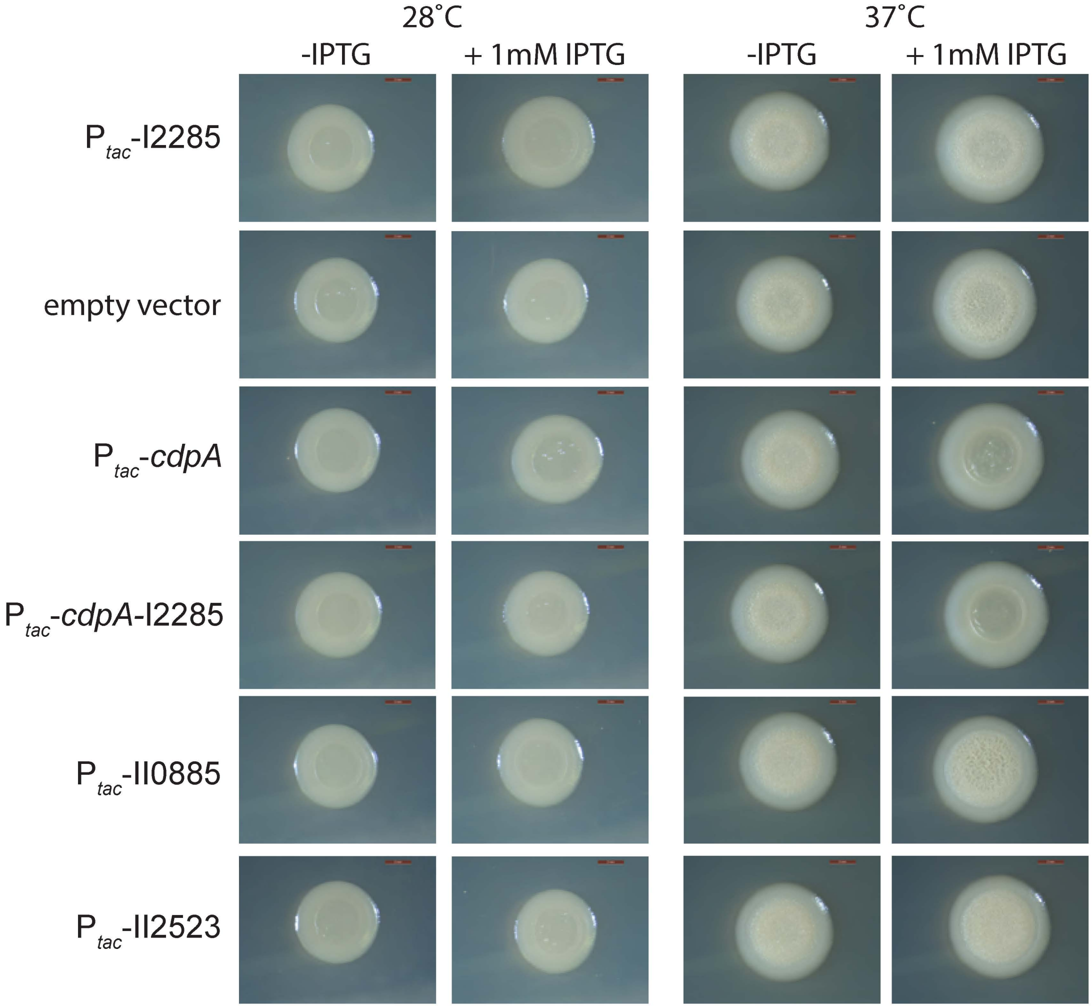

Fig S4A Inducible expression of c-di-GMP genes in the parental strain of *B. pseudomallei* Bp82. LB at 28°C and 37°C

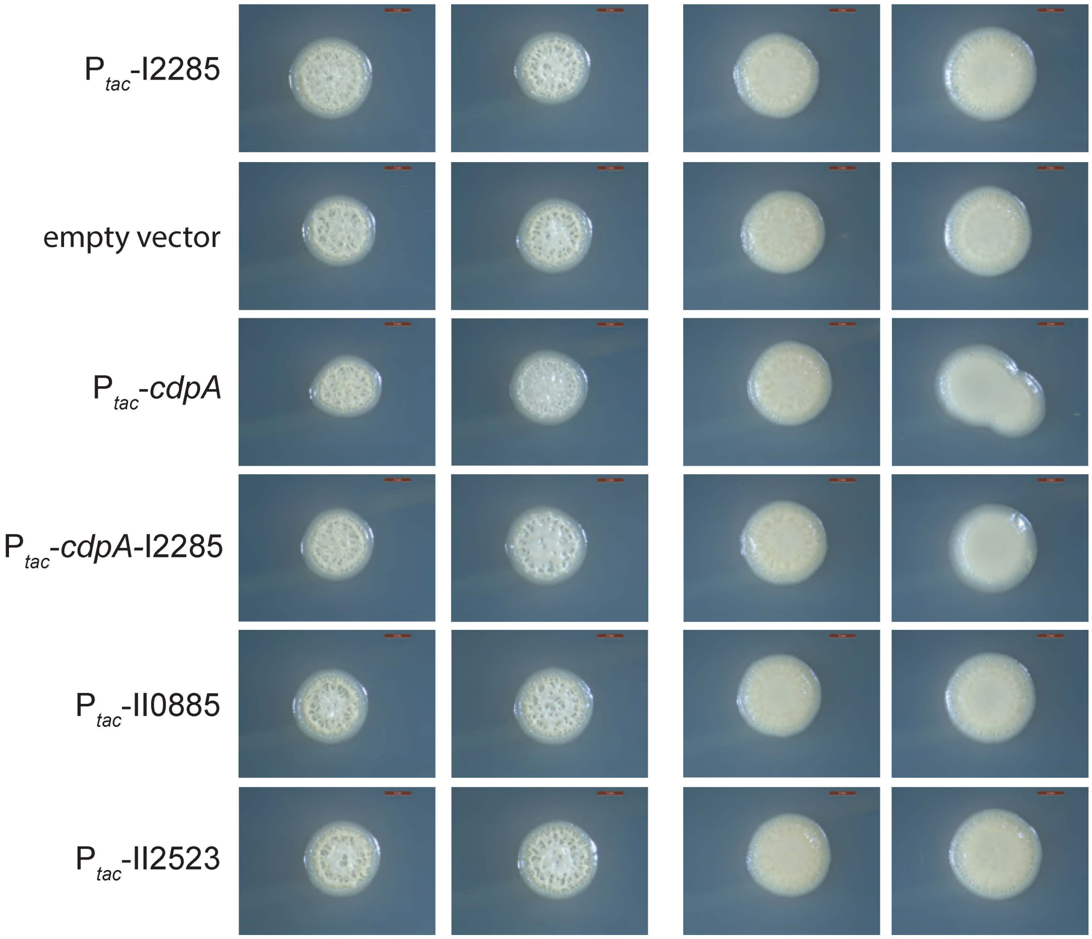

Fig S4B Inducible expression of c-di-GMP genes in the parental strain of *B. pseudomallei* Bp82. YEM at 28°C and 37°C

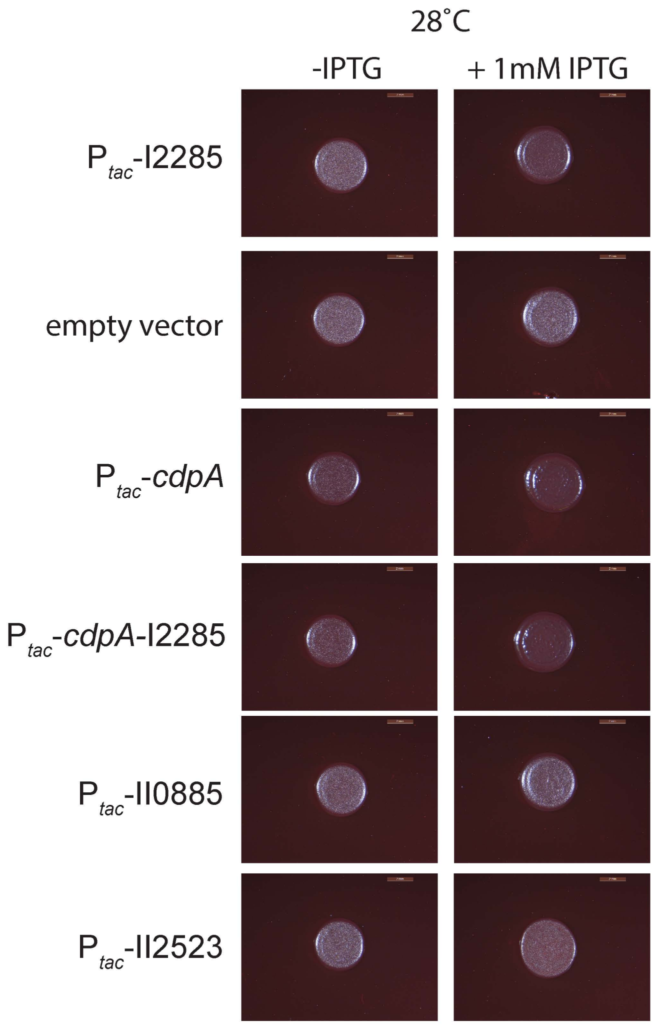

Fig S4C Inducible expression of c-di-GMP genes in the parental strain of *B. pseudomallei* Bp82. NAP-A at 28°C.

Fig S5

```
X. campestris gsmR          ---------MRILFVGDEASLPSDLVDYIAELGDNWQAQQVPDGNSAIEAAALSPFDAVI    51
P. aeruginosa PAO1 PA0267   --MSAAKKAPVILIADPDPWSRDLLGQLVLGVRCDARLVLCGDGGEALAHCRRRRFALIL    58
P. syringae PSPTO_0303      MTAVVLPAVPRVLIAESDPWVRETLSDLVLGVRADVELEMCTDGKQAVEWMKKQLPDLVI    60
B. pseudomallei I2285       ------------------------------------------------------------    0
C. jejuni CJ0248            ------------------------------------------------------------    0
G. sulfurreducens GSU2296   ------------------------------------------------------------    0
S. amazonensis Sama_3455    ------------------------------------------------------------    0

X. campestris gsmR          VAPVLPDLTAATLLGQIRTLRPDTIR-IALIDAQDGQRTPPARIIGVAHRFLPMPLAPEV    110
P. aeruginosa PAO1 PA0267   AELNLPQVDGFELLREARLRRSVAEQPFILISDRADQASVRAAVALAPTAYLVKPFQAEN    118
P. syringae PSPTO_0303      AARELPGIDGLSLLRGVRNLRRQPVIPFILISSRSDSASVREVLPLAPTAYLTKPLNTEG    120
B. pseudomallei I2285       ------------------------------------------------------------    0
C. jejuni CJ0248            ------------------------------------------------------------    0
G. sulfurreducens GSU2296   ------------------------------------------------------------    0
S. amazonensis Sama_3455    ------------------------------------------------------------    0

X. campestris gsmR          LLEAVTSLEELRD---------LLSNPRLRAAIGR----IEKLPSPPHLYLSLMHALEED    157
P. aeruginosa PAO1 PA0267   LMQRLRGLLLKGDEVVACPLPERDAGENLEAFLERARDSAEGAPLLADVRAASDRCLSAE    178
P. syringae PSPTO_0303      LRQRLESLLLEHRAPAPGEVPALTPGLTLTKFLDKRRDVADGAPLFVDVATAVKLSQTAN    180
B. pseudomallei I2285       -------------MPITAQLPRTALLDKLWARMNE----RGDFPLLSDALRATMAAMKND    43
C. jejuni CJ0248            -----------------------MIGDMNELLLKS----VEVLPPLPDTVSKLRKYVSEA    33
G. sulfurreducens GSU2296   -------------MDISGRDVE-TRLTLAREFMSG----VDELPTVPDIVLRIAGKLNDP    42
S. amazonensis Sama_3455    --------------------------MHSAALLQK----VDELPRLPKAIAELLDVVNNE    30
                                                            :          *   .

X. campestris gsmR          DG-ADAADIAKLIAGDPAIAAKVLQLCNSAFF-SGGRSITDLRTAVTRLGVATLRDLVLA    215
P. aeruginosa PAO1 PA0267   EH--PLRVLEAEFGRDPQITAGLIAAANSAAR-HVGPACQTLGQALRRLGLGHSLNLVLG    235
P. syringae PSPTO_0303      GI--DVALLEQELRNDPHITAVLIAAANSAAQ-HLGKPVQTMGGALGILGPVQGANIASG    237
B. pseudomallei I2285       EL--DFTALVRVVLSDFALTQKVLRLANSAMYMAFGGNITTVTRALMVLGMDAVGHLVVG    101
C. jejuni CJ0248            NSNIETMKVAEIISSDPLMTAKLLQLANSPYY-GFTREITTINQVITLLGVGNIINIVMA    92
G. sulfurreducens GSU2296   DVAI--DEVADLLLQDQVLTARVVHLANSPLY-SAARPISSIRDAVIYLGLDLLREAIFT    99
S. amazonensis Sama_3455    DSTV--KAVSEKLSHDPVLSARVLRLANSARF-GCSREVGTIDDAVVRLGMQTLRTLVIA    87
                                    :   .  *  ::  ::  .**            :  .:  **

X. campestris gsmR          SEVFSVQTLSPA--ERAA--MQRRALL----SSRLAAKVLPPTSAELGSTAALLADIGLL    267
P. aeruginosa PAO1 PA0267   FSLQRAIQLGEPLLAERAMHFWDLSQR-CADIAWELADALGAD-VERCYTAGLLHRLGDL    293
P. syringae PSPTO_0303      LAKKRMAVLTDDALLAQAHEFWTMSQR-TADYARILGGMLELD-VERCFCAGLLQSLGDL    295
B. pseudomallei I2285       LKLVDHFHHSA-PRRIDAKLELNRALL-SGCVARKLTEHVDLRAGEQAVVCTLMRQVGKL    159
C. jejuni CJ0248            DSIRDNFKIDVSPYGLNTQNFLKTCNEEATFIANWLNDEDKKL-SHLLVPCAMLLRLGIV    151
G. sulfurreducens GSU2296   CAIVDLFKTGKGPLNRST--LWAHSLG-VARIAKLIAERTGFLNPVNVYVAGLLHDVGEV    156
S. amazonensis Sama_3455    SAVVGAVPKVE-GFDLAD--FWGNTFE-VAIICQELAKRLGTL-PEEAFTCGILHSIGEL    142
                                                            .                 . ::  :* :

X. campestris gsmR          LPGVR---------------------DEREPPVEGDERLGHTEAGAYLLGLWGLPMPIIE    306
P. aeruginosa PAO1 PA0267   ALLRTLQ------DWCD------GGGALDEARLDELLGRFGASFGSALRARWRLPLELRR    341
P. syringae PSPTO_0303      AVLGCLQ------EWLL------AGGVLNEEVIKQSLEQYSAAFGSALRTRWRLPLELRE    343
B. pseudomallei I2285       LVVCYLD-----AEWEEIR-RRAAEHDDDEAACRAVLGVGYDEIGLEAASRWRLPELIRA    213
C. jejuni CJ0248            IFSNFLIQNHKDKDFL----AFLNKNENLALAENEFLGVDHISFLGFLLHRWNFDDVLIE    207
G. sulfurreducens GSU2296   FINFF-----RGKEFSQVVTLVDEEKITFGQAEERLFGTSHCEVGFALAKRWSLNEFICD    211
S. amazonensis Sama_3455    LIVNG-----DPAVAAT-ISAAVADGADRNLMEKELLGYDNAEIGALLAQSWKFTPHLVK    196
                                                                               * :   :
```

```
X. campestris gsmR          AVAFHRHPQRSSLRSFWV-TGAVHVATALASGETVDEEYL----TKVGVINRLPNWREQA   361
P. aeruginosa PAO1 PA0267   LIAAAYQFGG-VLSREEL---ILSLATQAA----------------SLPEDDA-EQLAES   380
P. syringae PSPTO 0303      LIAAVYQYNTGIFTREVL---AMNLAGQMA----------------RLGEEESVTPLIKT   384
B. pseudomallei I2285       GMTRFDANDGAPHEVQWL-RAVSHCSTDVAT----------ALTSTHAQQRDARIAALAH   262
C. jejuni CJ0248            SICFVRTPHAAREKVKKSAYA-LAITDHLFA----PHDGSSPFNAK------AAVALLKE   256
G. sulfurreducens GSU2296   TILYHHD-IEAVPYKQAAIVAMVAFADEYCTLRRLGFEGHKPVDSVRTLLENHPSWGVIR   270
S. amazonensis Sama_3455    GIQFQNHPKSAEPYSKLA--GMLAMAKQIAA------DWD------KIPDDERTSWLAQI   242
                             :                       :

X. campestris gsmR          DT--LMGLAEA-------------------------------------------------   370
P. aeruginosa PAO1 PA0267   KAARMLGLDGERLAKLLQ------------P-----------------------------   399
P. syringae PSPTO_0303      KSAKLLKLSVGDLQRLRKKLTGVTDPSLLIPLPVEPEAVADAVEEDE-----------HD   433
B. pseudomallei I2285       RFHGVLGTDADELADIAASLSREETSDTVMREIVELRANADKIARGASTPQACLEAGLAD   322
C. jejuni CJ0248            AK--TQGINF-DLNNLLSKLPNKA------KENLNKED----------------------   285
G. sulfurreducens GSU2296   RS--LGGSDF-DEKLIVA---ELD------SSIVEIRAAVDE------------------   300
S. amazonensis Sama_3455    NI--LAGIKV-DLGGLAEKLAKMH------GQGMEMGKQLA-------------------   274


X. campestris gsmR          ------------------------------------------------------------   370
P. aeruginosa PAO1 PA0267   ------------------------------------------------------------   399
P. syringae PSPTO_0303      LLDLTPEAPIQEPAALEDPGDAVQGVTKK-------------------------------   462
B. pseudomallei I2285       LRAL-PSEHVLGPVLALASESVLAGLAFTRTVMFVRHDDGVFAARLGFGPNVDAALERLR   381
C. jejuni CJ0248            ------------------------------------------------------------   285
G. sulfurreducens GSU2296   LFLL--------------------------------------------------------   304
S. amazonensis Sama_3455    ------------------------------------------------------------   274


X. campestris gsmR          ------------------------------------------------------------   370
P. aeruginosa PAO1 PA0267   ------------------------------------------------------------   399
P. syringae PSPTO_0303      ------------------------------------------------------------   462
B. pseudomallei I2285       FHETFEPDVFHLAITNSVGIFIEHARDPKMTKRLPAWYLDAFDDARAFVLLPVIAERNAV   441
C. jejuni CJ0248            ------------------------------------------------------------   285
G. sulfurreducens GSU2296   ------------------------------------------------------------   304
S. amazonensis Sama_3455    ------------------------------------------------------------   274


X. campestris gsmR          -------------------------------------------370
P. aeruginosa PAO1 PA0267   -------------------------------------------399
P. syringae PSPTO_0303      -------------------------------------------462
B. pseudomallei I2285       ALLYGDWALGQPARKITPQEMGVLNELAAELGRFFCGAQGDDR484
C. jejuni CJ0248            -------------------------------------------285
G. sulfurreducens GSU2296   -------------------------------------------304
S. amazonensis Sama_3455    -------------------------------------------274
```

Fig S5 *B. pseudomallei* I2285 alignment with other predicted HDOD containing proteins from diverse bacterial strain. Protein alignment was done using clustal omega (Clustal O (1.2.4) multiple sequence alignment. *P. syringae* = *P. syringae* pv. tomato DC3000. Residues in red and underlined were targeted for site-directed mutagenesis. Bacterial strains, corresponding gene loci, and NCBI accession numbers shown in parentheses for the predicted HDOD containing proteins are denoted for each sequence: *X. campestris gsmR* (WP_011038414.1), *P. aeruginosa* PAO1 PA0267 (AAG03656.1), *P. syringae* PSPTO_0303 (AAO53848.1), *B. pseudomallei* Bp1026b_I2285 (AFI66892.1), *C. jejuni* CJ0248 (CAL34402.1), *G. sulfurreducens* GSU2296 (AAR35672.1), *S. amazonensis* Sama_3455(ABM01658.1).

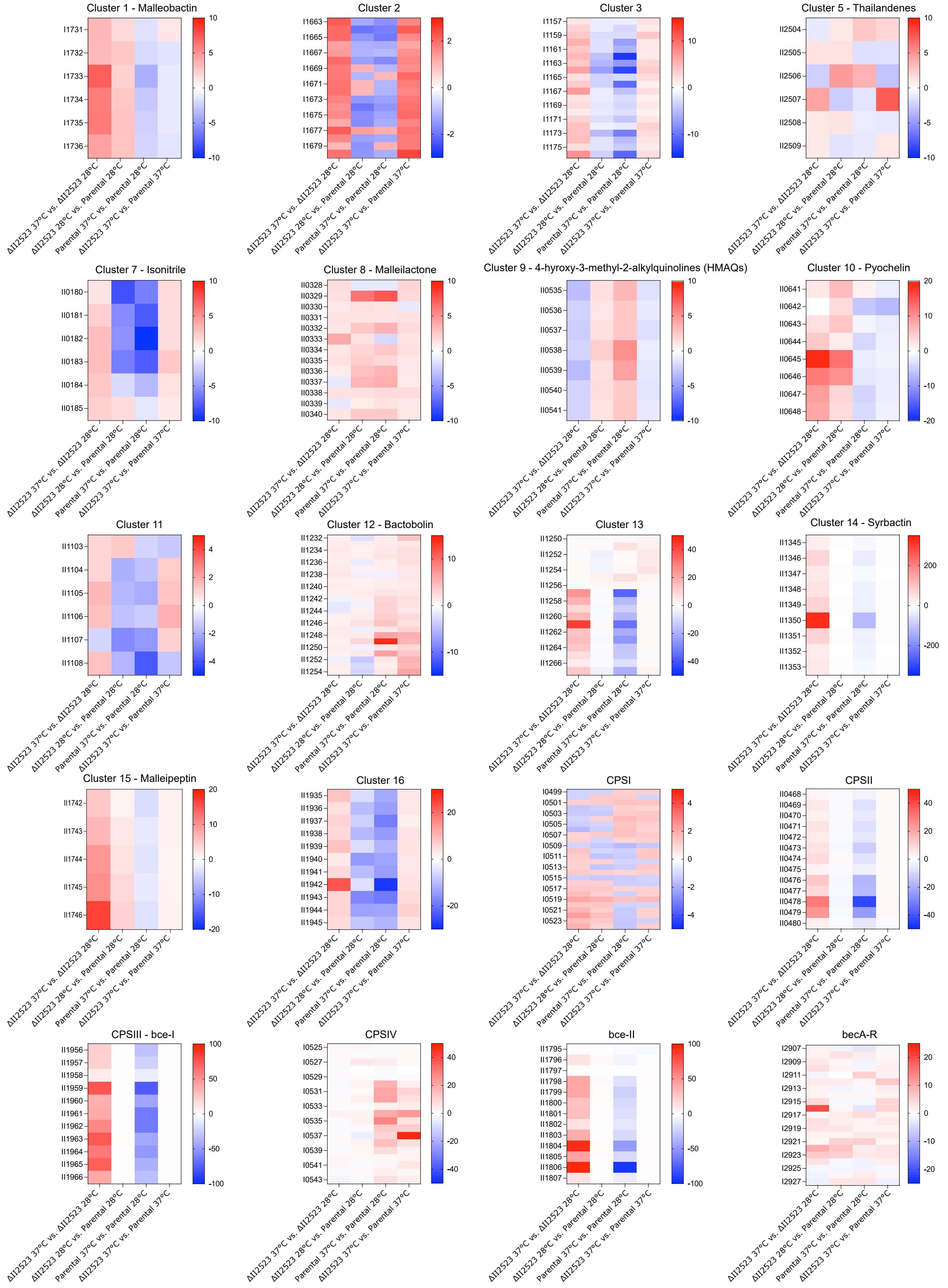

Fig S6 Heatmaps of complete predicted clusters show differential expression patterns of ΔII2523 in four pairwise comparisons. Raw fold change values for all predicted clusters described in this study were input from DESeq2. Color scales are consistent throughout (red being highest, white being neutral, and blue being lowest), although ranges vary to accommodate individual panels' fold change differences.

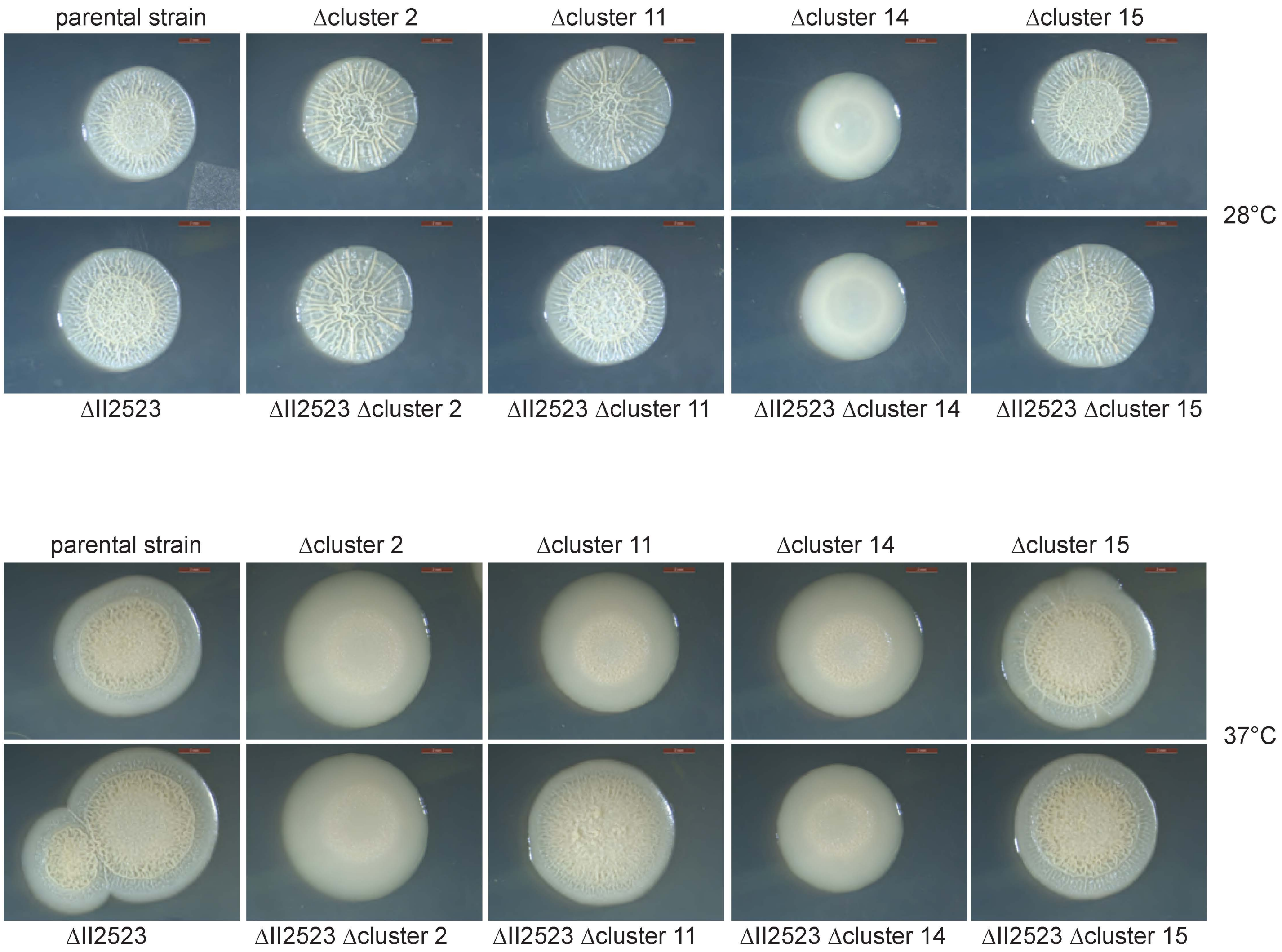

Fig S7 Colony morphology of NRPS/PKS mutants in the Bp82 parental strain or Bp82 ΔII2523 backgrounds grown on LB media at 28°C or 37°C. Images were taken after four days of growth. Scale bar represents 2mm.

Supplemental Table 1. Microbial strains, plasmids, and primers used in this study.			
**Species**	**Genotype**	**Source**	**Purpose**
E. coli RHO3	SM10 (λ pir) Δasd::FRT ΔaphA::FRT	(1)	Strain used for conjugation of mobilizable plasmids that allows for donor counter-selection.
B. subtilis 3610	*B. subtilis* ssp. *subtilis* NCIB3610	Bacillus Genetic Stock Center	Wild-type undomesticated strain
R. solani	AG2-2 IIIB-R9	This study	Gift from J. Leach laboratory, Colorado State University
B. pseudomallei	1026b	(2)	Wild-type BSL3 select agent
B. pseudomallei	1026b Δ*cdpA*	This study	In-frame deletion of *cdpA* (Bp1026b_I2284)
B. pseudomallei	1026b Δ*cdpA*-I2285	This study	In-frame deletion of *cdpA* and Bp1026b_I2285
B. pseudomallei	1026b ΔI2285	This study	In-frame deletion of Bp1026b_I2285
B. pseudomallei	1026b Δ*cdpA*-I2285 ΔII2523	This study	In-frame deletion of *cdpA,* Bp1026b_I2285 and Bp1026b_II2523
B. pseudomallei	1026b ΔII0885	This study	In-frame deletion of Bp1026b_II0885
B. pseudomallei	1026b Δ*cdpA* ΔII0885	This study	In-frame deletion of *cdpA* and Bp1026b_II0885
B. pseudomallei	1026b ΔI2285 ΔII0885	This study	In-frame deletion of Bp1026b_I2285 and Bp1026b_II0885
B. pseudomallei	1026b Δ*cdpA*-I2285 ΔII0885	This study	In-frame deletion of *cdpA* Bp1026b_I2285 and Bp1026b_II0885
B. pseudomallei	1026b ΔII2523	This study	In-frame deletion of Bp1026b_II2523
B. pseudomallei	1026b Δ*cdpA* ΔII2523	This study	In-frame deletion of *cdpA* and Bp1026b_II2523
B. pseudomallei	1026b ΔI2285 ΔII2523	This study	In-frame deletion of Bp1026b_I2285 and Bp1026b_II2523
B. pseudomallei	1026b ΔII0885 ΔII2523	This study	In-frame deletion of Bp1026b_II2523 and Bp1026b_II0885

B. pseudomallei	1026b Δ*cdpA*-I2285 ΔII0885 ΔII2523	This study	In-frame deletion of *cdpA,* Bp1026b_I2285, Bp1026b_II0885, and Bp1026b_II2523
B. pseudomallei	Bp82	(3)	Δ*purM* derivative of Bp1026b, select agent excluded BSL-2 compliant strain
B. pseudomallei	Bp82 Δ*cdpA*	This study	In-frame deletion of *cdpA* (Bp1026b_I2284) in Bp82
B. pseudomallei	Bp82 Δ*cdpA*-I2285	This study	In-frame deletion of *cdpA* and Bp1026b_I2285 in Bp82
B. pseudomallei	Bp82 ΔI2285	This study	In-frame deletion of Bp1026b_I2285 in Bp82
B. pseudomallei	Bp82 ΔcdpA-I2285 ΔII2523	This study	In-frame deletion of *cdpA,* Bp1026b_I2285 and Bp1026b_II2523 in Bp82
B. pseudomallei	Bp82 ΔII0885	This study	In-frame deletion of Bp1026b_II0885 in Bp82
B. pseudomallei	Bp82 Δ*cdpA* ΔII0885	This study	In-frame deletion of *cdpA* and Bp1026b_II0885 in Bp82
B. pseudomallei	Bp82 ΔI2285 ΔII0885	This study	In-frame deletion of Bp1026b_I2285 and Bp1026b_II0885 in Bp82
B. pseudomallei	Bp82 Δ*cdpA*-I2285 ΔII0885	This study	In-frame deletion of *cdpA,* Bp1026b_I2285 and Bp1026b_II0885 in Bp82
B. pseudomallei	Bp82 ΔII2523	This study	In-frame deletion of Bp1026b_II2523 in Bp82
B. pseudomallei	Bp82 Δ*cdpA* ΔII2523	This study	In-frame deletion of *cdpA* and Bp1026b_II2523 in Bp82
B. pseudomallei	Bp82 ΔI2285 ΔII2523	This study	In-frame deletion of Bp1026b_I2285 and Bp1026b_II2523 in Bp82
B. pseudomallei	Bp82 ΔII0885 ΔII2523	This study	In-frame deletion of Bp1026b_II2523 and Bp1026b_II0885 in Bp82

B. pseudomallei	Bp82 Δ*cdpA*-I2285 ΔII0885 ΔII2523	This study	In-frame deletion of *cdpA*-Bp1026b_I2285, Bp1026b_II0885, and Bp1026b_II2523 in Bp82
B. pseudomallei	Bp82 P_{tac}-EV (empty vector)	This study	Bp82 with integrated P_{tac}-empty vector control
B. pseudomallei	Bp82 P_{tac}-*cdpA*	This study	Conditional expression of full-length Bp1026b_I2284 under the control of the P_{tac}-promoter
B. pseudomallei	Bp82 P_{tac}-I2285	This study	Conditional expression of full-length Bp1026b_I2285 under the control of the P_{tac}-promoter
B. pseudomallei	Bp82 P_{tac}-*cdpA*-I2285	This study	Conditional expression of full-length Bp1026b_I2284 and Bp1026b_I2285 under the control of the P_{tac}-promoter
B. pseudomallei	Bp82 P_{tac}-II0885	This study	Conditional expression of full-length Bp1026b_II0884 under the control of the P_{tac}-promoter
B. pseudomallei	Bp82 P_{tac}-II2523	This study	Conditional expression of full-length Bp1026b_II2523 under the control of the P_{tac}-promoter
B. pseudomallei	Bp82 Δ*cdpA* P_{tac}-EV (empty vector)	This study	In-frame deletion of *cdpA* (Bp1026b_I2284) with integrated $_{Ptac}$-empty vector control
B. pseudomallei	Bp82 Δ*cdpA* P_{tac-}*cdpA*	This study	In-frame deletion of *cdpA* (Bp1026b_I2284) complemented with full-length Bp1026b_I2284 under the control of the P_{tac}-promoter
B. pseudomallei	Bp82 Δ*cdpA* P_{tac}-*cdpA*^{S72A}	This study	In-frame deletion of *cdpA* (Bp1026b_I2284) complemented with full-length Bp1026b_I2284^{S72A} under the control of the P_{tac}-promoter

B. pseudomallei	Bp82 Δ*cdpA* P_{tac}-*cdpA*^{AAL}	This study	In-frame deletion of *cdpA* (Bp1026b_I2284) complemented with full-length Bp1026b_I2284^{AAL} under the control of the P_{tac}-promoter
B. pseudomallei	Bp82 ΔII2523 EV	This study	In-frame deletion of Bp1026b_II2523 with integrated $_{Ptac}$-empty vector control
B. pseudomallei	Bp82 ΔII2523 P_{tac}-II2523	This study	In-frame deletion of Bp1026b_II2523 complemented with full-length Bp1026b_II2523 under the control of the P_{tac}-promoter
B. pseudomallei	Bp82 ΔII2523 P_{tac}-II2523^{N32A}	This study	In-frame deletion of Bp1026b_II2523 complemented with full-length Bp1026b_II2523^{N32A} under the control of the P*tac*-promoter
B. pseudomallei	Bp82 ΔI2285 P_{tac}-EV	This study	In-frame deletion of Bp1026b_I2285 with integrated $_{Ptac}$-empty vector control
B. pseudomallei	Bp82 ΔI2285 P_{tac}-I2285	This study	In-frame deletion of Bp1026b_I2285 complemented with full-length Bp1026b_I2285 under the control of the Ptac-promoter
B. pseudomallei	Bp82 ΔI2285 P_{tac}-II2523^{H179A}	This study	In-frame deletion of Bp1026b_I2285 complemented with full-length Bp1026b_I2285^{H179A} under the control of the P_{tac}-promoter
B. pseudomallei	Bp82 ΔI2285 P_{tac}-II252 ^{H262A}	This study	In-frame deletion of Bp1026b_I2285 complemented with full-length Bp1026b_I2285^{H262A} under the control of the P_{tac}-promoter
B. pseudomallei	Bp82 ΔI2285 P_{tac}-I2285 ^{H358A}	This study	In-frame deletion of Bp1026b_I2285 complemented with full-length Bp1026b_I2285^{H358A} under the control of the P_{tac}-promoter

B. pseudomallei	Bp82 ΔI2285 P_{tac}-I2285 ^{Y194A}	This study	In-frame deletion of Bp1026b_I2285 complemented with full-length Bp1026b_I2285^{Y194A} under the control of the P_{tac}-promoter
B. pseudomallei	Bp82 ΔI2285 P_{tac}-I2285 ^{W205A}	This study	In-frame deletion of Bp1026b_I2285 complemented with full-length Bp1026b_I2285^{W205A} under the control of the P_{tac}-promoter
B. pseudomallei	Bp82 ΔI2285 P_{tac}-I2285 $^{W205A\ Y194A}$	This study	In-frame deletion of Bp1026b_I2285 complemented with full-length Bp1026b_I2285$^{W205A\ Y194A}$ under the control of the P_{tac}-promoter
B. pseudomallei	Bp82 Δcluster 2	This study	Disruption of cluster 2 (unknown NRPS) using pKOB
B. pseudomallei	Bp82 Δcluster 11	This study	Disruption of cluster 11 (unknown NRPS) using pKOC
B. pseudomallei	Bp82 Δcluster 14	This study	Disruption of cluster 14 (syrbactin) using pKOD
B. pseudomallei	Bp82 Δcluster 15	This study	Disruption of cluster 15 (malleipeptin) using pKOE
B. pseudomallei	Bp82 ΔII2523 Δcluster 2	This study	Disruption of Cluster 2 (unknown NRPS) in Bp82 ΔII2523 using pKOB
B. pseudomallei	Bp82 ΔII2523 Δcluster 11	This study	Disruption of cluster 11 (unknown NRPS) in Bp82 ΔII2523 using pKOC
B. pseudomallei	Bp82 ΔII2523 Δcluster 14	This study	Disruption of cluster 14 (syrbactin) in Bp82 ΔII2523 using pKOD
B. pseudomallei	Bp82 ΔII2523 Δcluster 15	This study	Disruption of cluster 15 (malleipeptin) in Bp82 ΔII2523 using pKOE
Pseudomonas aeruginosa PAO1	*P. aeruginosa* PAO1	(4)	Wild type *Pseudomonas aeruginosa*
P. aeruginosa PAO1	PAO1 (EV)	This study	Bp82 with integrated P_{tac}-empty vector control

P. aeruginosa PAO1	PAO1 P_{tac}-*cdpA*	This study	Conditional expression of full-length Bp1026b_I2284 under the control of the P_{tac}-promoter
P. aeruginosa PAO1	PAO1 P_{tac}-I2285	This study	Conditional expression of full-length Bp1026b_I2285 under the control of the P_{tac}-promoter
P. aeruginosa PAO1	PAO1 P_{tac}-*cdpA*-I228*5*	This study	Conditional expression of full-length Bp1026b_I2284 (*cdpA*) and Bp1026b_I2285 under the control of the P_{tac}-promoter
P. aeruginosa PAO1	PAO1 P_{tac}-II0885	This study	Conditional expression of full-length Bp1026b_II0885 under the control of the P_{tac}-promoter
P. aeruginosa PAO1	PAO1 P_{tac}-II2523	This study	Conditional expression of full-length Bp1026b_I2523 under the control of the P_{tac}-promoter
P. aeruginosa PAO1 Δ*wspF*	Δ*wspF*	(5)	Δ*wspF* strain is hyperbiofilm forming, swim defecient, rugose colony forming, and elevated in the production of c-di-GMP
P. aeruginosa PAO1 Δ*wspF*	Δ*wspF* EV	This study	Δ*wspF* with integrated P_{tac}-empty vector control
P. aeruginosa PAO1 Δ*wspF*	Δ*wspF* P_{tac}-*cdpA*	This study	Conditional expression of full-length Bp1026b_I2284 under the control of the P_{tac}-promoter
P. aeruginosa PAO1 Δ*wspF*	Δ*wspF* P_{tac}-I2285	This study	Conditional expression of full-length Bp1026b_I2285 under the control of the P_{tac}-promoter
P. aeruginosa PAO1 Δ*wspF*	Δ*wspF* P_{tac}-*cdpA*-I228*5*	This study	Conditional expression of full-length Bp1026b_I2284 *(cdpA*) and Bp1026b_I2285 under the control of the P_{tac}-promoter

P. aeruginosa PAO1 Δ*wspF*	Δ*wspF* P_{tac}-II0885	This study	Conditional expression of full-length Bp1026b_II0885 under the control of the P_{tac}-promoter
P. aeruginosa PAO1 Δ*wspF*	Δ*wspF* P_{tac}-II2523	This study	Conditional expression of full-length Bp1026b_I2523 under the control of the P_{tac}-promoter
Plasmids			
	pFLPe2	(6)	Flp recombinase for kanamycin cassette excision of mini-Tn7T-Km-LAC insertions
	pTNS3	(6)	Helper plasmid
	pEXKm5	(1)	Allelic exchange vector for generating markerless deletions
	pEXKm5 Δ*cdpA*	This study	Allelic exchange vector for generating markerless Δ*cdpA* deletions
	pEXKm5 ΔI2285	This study	Allelic exchange vector for generating markerless ΔI2285 deletions
	pEXKm5 Δ*cdpA*-I2285	This study	Allelic exchange vector for generating markerless Δ*cdpA*-I2285 deletions
	pEXKm5 ΔII2523	This study	Allelic exchange vector for generating markerless ΔII2523 deletions
	pEXKm5 ΔII0885	This study	Allelic exchange vector for generating markerless ΔII0885 deletions
	pUC18T-mini-Tn7T-Km-LAC	(7)	Tn7 integration vector for IPTG-mediated conditional expression
	pUC18T-mini-Tn7T-Km-LAC::*cdpA*	This study	Tn7 integration vector for IPTG-mediated conditional expression of *cdpA* (Bp1026b_I2284)
	pUC18T-mini-Tn7T-Km-LAC::I2285	This study	Tn7 integration vector for IPTG-mediated conditional expression of Bp1026b_I2285 in Bp82

	pUC18T-mini-Tn7T-Km-LAC::cdpA-I2285	This study	Tn7 integration vector for IPTG-mediated conditional expression of *cdpA* and Bp1026b_I2285 in Bp82
	pUC18T-mini-Tn7T-Km-LAC::II2523	(8)	Tn7 integration vector for IPTG-mediated conditional expression of Bp1026b_II2523
	pUC18T-mini-Tn7T-Km-LAC::II0885	This study	Tn7 integration vector for IPTG-mediated conditional expression of Bp1026b_II0885
	pKOB	(9)	Gift from D. DeShazer (USARMID) pEXKm5 deletion plasmid for disrupting cluster 2 I1663-I1679
	pKOC	(9)	Gift from D. DeShazer (USARMID) pEXKm5 deletion plasmid cluster 11 II1089-II1108
	pKOD	(9)	Gift from D. DeShazer (USARMID) pEXKm5 deletion plasmid for disrupting cluster 14 II1345-II1353-syrbactin
	pKOE	(9)	Gift from D. DeShazer (USARMID) pEXKm5 deletion plasmid for disrupting cluster 15 II1742-II1746-malleipeptin
Primers for construction of complementation vectors			
	NNNCCCGGGAGGAGGATATTCATGGAAGCCATCAGGAACAA	This study	pUC18T-mini-Tn7T-Km-LAC::*cdpA*
	NNNAAGCTTTCATGCGGTGGCGTGCAGAT	This study	pUC18T-mini-Tn7T-Km-LAC::*cdpA*
	NNNCCCGGGAGGAGGATATTCATGCCTATCACCGCACAACTGCC	This study	pUC18T-mini-Tn7T-Km-LAC::I2285
	NNNAAGCTTCTACCGATCGTCGCCCTGCGCG	This study	pUC18T-mini-Tn7T-Km-LAC::I2285

	NNNCCCGGGAGGAGGATATTCATGGAAGCCATCAGGAACAA	This study	pUC18T-mini-Tn7T-Km-LAC::*cdpA*-I2285
	NNNAAGCTTCTACCGATCGTCGCCCTGCGCG	This study	pUC18T-mini-Tn7T-Km-LAC::*cdpA*-I2285
	NNNCCCGGGAGGAGGATATTCATGCACGGCACCTACAACCT	This study	pUC18T-mini-Tn7T-Km-LAC::II0885
	NNNCCCGGGTCATGCCGGCGCCTCGGAGT	This study	pUC18T-mini-Tn7T-Km-LAC::II0885
Soeing PCR primers			
Bp1026b_I2284 (*cdpA*) deletion			
	TGATGCCCGCGCGCGCCGGTCATCTGCACGCCACCGCATGAC	This study	Amplification of left flanking region of Bp1026b_I2284 (*cdpA*)
	CTCGAGGCCGATCTCGTCGTAGC	This study	Amplification of left flanking region of Bp1026b_I2284 (*cdpA*)
	CCCGGGAACTTGCTGCGGCATTCGCTG	This study	Amplification of right flanking region of Bp1026b_I2284 (*cdpA*)
	TCATGCGGTGGCGTGCAGATGACCGGCGCGCGCGGGCATCAG	This study	Amplification of right flanking region of Bp1026b_I2284 (*cdpA*)
	GCTTCCTGTACGTGTCCGATGC	This study	Internal PCR confirmation
	TTGACGGCCACGTACTGCAG	This study	Internal PCR confirmation
	GAATGATCTTCGCGTCCGGA	This study	External PCR confirmation
	GGTTCAGTTCGAGCTTCGCGT	This study	External PCR confirmation
Bp1026b_I2285 deletion			
	NNNCCCGGGGTCGGCATCGCGATCTATCC	This study	Amplification of left flanking region of Bp1026b_I2285
	TCGAACGCTACCGATCGTCTTGTGCGGTGATAGGCATACG	This study	Amplification of left flanking region of Bp1026b_I2285
	NNNGAATTCGCACAACGACATCAGCGA	This study	Amplification of right flanking region of Bp1026b_I2285

	CGTATGCCTATCACCGCACAAGACGATCGGTAGCGTTCGA	This study	Amplification of right flanking region of Bp1026b_I2285
	GGATGAACGAGCGCGGCGAT	This study	Internal PCR confirmation
	GAAAGCGCAACCGTTCGAGC	This study	Internal PCR confirmation
	GCATTCGCTGACCGAAAACTTCGTCA	This study	External PCR confirmation
	TCGCATGGAGCATTGACGGC	This study	External PCR confirmation
Bp1026b_I2284 (*cdpA*)-Bp1026b_I2285 deletion			
	NNNCCCGGGAACTTGCTGCGGCATTCGCTG	This study	Amplification of left flanking region of Bp1026b_I2284 (*cdpA*)-Bp1026b_I2285
	TCGAACGCTACCGATCGTCACCGGCGCGCGCGGGCATCAG	This study	Amplification of left flanking region of Bp1026b_I2284 (*cdpA*)-Bp1026b_I2285
	CTGATGCCCGCGCGCGCCGGTGACGATCGGTAGCGTTCGA	This study	Amplification of right flanking region of Bp1026b_I2284 (*cdpA*)-Bp1026b_I2285
	NNNCTCGAGGCACAACGACATCAGCGA	This study	Amplification of right flanking region of Bp1026b_I2284 (*cdpA*)-Bp1026b_I2285
	GGATGAACGAGCGCGGCGAT	This study	Internal PCR confirmation ΔI2284
	GAAAGCGCAACCGTTCGAGC	This study	Internal PCR confirmation ΔI2284
	GCATTCGCTGACCGAAAACTTCGTCA	This study	External PCR confirmation ΔI2284
	TCGCATGGAGCATTGACGGC	This study	External PCR confirmation ΔI2284
	GGATGAACGAGCGCGGCGAT	This study	Internal PCR confirmation ΔI2285
	GAAAGCGCAACCGTTCGAGC	This study	Internal PCR confirmation ΔI2285
	GCATTCGCTGACCGAAAACTTCGTCA	This study	External PCR confirmation ΔI2285
	TCGCATGGAGCATTGACGGC	This study	External PCR confirmation ΔI2285

Bp1026b_II2523 deletion			
	GAACGAAGCATCGTCCTCCCA	This study	Amplification of left flanking region of Bp1026b_II2523
	TCAACCGAACGCAACCGAGCACAGCGAGGAAAGCAGGTTCAT	This study	Amplification of left flanking region of Bp1026b_II2523
	TCGTTATCGTCCACCACGGGA	This study	Amplification of right flanking region of Bp1026b_II2523
	ATGAACCTGCTTTCCTCGCTGTGCTCGGTTGCGTTCGGTTGA	This study	Amplification of right flanking region of Bp1026b_II2523
	GCTCGACCGCGAGATGAGGGT	This study	Internal PCR confirmation
	CAGCAGCAGCGCGAATTCCTC	This study	Internal PCR confirmation
	GCGTGCGTATCGTCGATCCTC	This study	External PCR confirmation
	GAGAAGATCGCGAAGCTCGTC	This study	External PCR confirmation
Bp1026b_II0885 deletion			
	TCCATGCACGGCACCTACAACCCGGCATGACGGTGACGC	This study	Amplification of left flanking region of Bp1026b_II0885
	GCGTCACCGTCATGCCGGGTTGTAGGTGCCGTGCATGGA	This study	Amplification of left flanking region of Bp1026b_II0885
	GCTTGAGGCCGTCGTTGTCCTG	This study	Amplification of right flanking region of Bp1026b_II0885
	NNNGAATTCATTGCCCGCGCTCAGCTGC	This study	Amplification of right flanking region of Bp1026b_II0885
	CGATCACCGGCCTGTCGCT	This study	Internal PCR confirmation
	ATGTCGCCGTCTCGCACG	This study	Internal PCR confirmation
	GAGTGCTTTTCGGTGACGGGC	This study	External PCR confirmation
	GGTGTGGCGAGTATCGGCAAG	This study	External PCR confirmation
QPCR primers			
	TCGCTCAACAGAACCTCAAC	This study	fliC I3555-F

	GTTGATCTGCGTTTGCATCC	This study	fliC I3555-R
	AAGCTGGCTGGAGCAAA	This study	cdpA I2284-F
	GCAGATAGTCGCGGTGATAA	This study	cdpA I2284-R
	GTTCGAGCCGGACGTATTT	This study	I2285 qPCR-F
	GCAGACGCTTGGTCATCTT	This study	I2285 qPCR-R
	GTCGACGTCTTCAACCACTATC	This study	II1799 qPCR-F
	CAGCTTGATGATGTCCTCTTCC	This study	II1799 qPCR-R
	CACCACATCAGCGACGTATC	This study	I2928 qPCR-F
	GCACGTACAGGCGGAAAT	This study	I2928 qPCR-R
	GAGCAGATGCAATCGGAGAT	This study	I2235 qPCR-F
	GGCCGTAGTTGTCGTTGAT	This study	I2235 qPCR-R
	GAATTGCTGCGTCGCTTTC	This study	I1579 qPCR-F
	GGCTCACGCAGATTCAGTT	This study	I1579 qPCR-R
	ATCGGCGACGACGTACA	This study	II0478 qPCR-F
	GCAGCCGTTCCCGATTT	This study	II0478 qPCR-R
	CAGATTCACCGGATCGTCAC	This study	cpsIII II1965-F
	CTCGATGACCTCCTGATTGAAG	This study	cpsIII II1965-R
	CAATCTCGCCAATCGCTTTC	This study	I0530 qPCR-F
	GAATGCCGGACGACGAATA	This study	I0530 qPCR-R
Quickchange mutagenesis primers			
	GGATCAGCGCCGCGACGCCGACC	This study	Primers for CdpA EAL to AAL site-directed mutagenesis
	GGTCGGCGTCGCGGCGCTGATCC	This study	Primers for CdpA EAL to AAL site-directed mutagenesis
	CAGGCTGGCATCGGCCACGTACAGGAAGC	This study	Primers for CdpA S72A site-directed mutagenesis
	GCTTCCTGTACGTGGCCGATGCCAGCCTG	This study	Primers for CdpA S72A site-directed mutagenesis

	CTCGTCGTCGTCAGCCTCGGCCGCGCGC	This study	Primers for I2285 H179A site-directed mutagenesis
	GCGCGCGGCCGAGGCTGACGACGACGAG	This study	Primers for I2285 H179A site-directed mutagenesis
	GCCGCGCTCGCGGCCCGCTTTCACGG	This study	Primers for I2285 H262A site-directed mutagenesis
	CCGTGAAAGCGGGCCGCGAGCGCGGC	This study	Primers for I2285 H262A site-directed mutagenesis
	CACGCCGTCGTCGGCCCGCACGAACATC	This study	Primers for I2285 H358A site-directed mutagenesis
	GATGTTCGTGCGGGCCGACGACGGCGT	This study	Primers for I2285 H358A site-directed mutagenesis
	GCCGATCTCGTCGGCGCCGACGCCGAGC	This study	Primers for I2285 Y194A site-directed mutagenesis
	GCTCGGCGTCGGCGCCGACGAGATCGG	This study	Primers for I2285 Y194A site-directed mutagenesis
	CCGGCAGCCGCGCGCGCGATGCCG	This study	Primers for I2285 W205A site-directed mutagenesis
	CGGCATCGCGCGCGCGGCTGCCGG	This study	Primers for I2285 W205A site-directed mutagenesis
	GTCCTGCATGAACCGGGCCCACATGAGTACCCTC	This study	Primers for II2523 PAS site-directed mutagenesis
	GAGGGTACTCATGTGGGCCCGGTTCATGCAGGAC	This study	Primers for II2523 PAS site-directed mutagenesis
clusterBdel-F	GCGGTGCATTTCTTCGAC	(9)	Primers for verification of cluster 2 disruption
clusterBdel-R	CATCGGCGATCACGAATC	(9)	Primers for verification of cluster 2 disruption
clusterCdel-F	GAAATTCCATGTGCGGTTTT	(9)	Primers for verification of cluster 11 disruption

clusterCdel-R	ACGTCGGGATTCTGATTGAC	(9)	Primers for verification of cluster 11 disruption
clusterDdel-F	AGTCGCTCCAGGTGGTTTC	(9)	Primers for verification of cluster 14 disruption
clusterDdel-R	GCGGATCTTGATCTGGTTGT	(9)	Primers for verification of cluster 14 disruption
clusterEdel-F	GTGAATGGGCGAGAGCAG	(9)	Primers for verification of cluster 15 disruption
clusterEdel-R	ATGTAGGCCGGCAGATGTC	(9)	Primers for verification of cluster 15 disruption

Supplemental references

1. Lopez CM, Rholl DA, Trunck LA, Schweizer HP. 2009. Versatile dual-technology system for markerless allele replacement in *Burkholderia pseudomallei*. Appl Environ Microbiol 75:6496-503.
2. DeShazer D, Brett PJ, Carlyon R, Woods DE. 1997. Mutagenesis of *Burkholderia pseudomallei* with Tn5-OT182: isolation of motility mutants and molecular characterization of the flagellin structural gene. J Bacteriol 179:2116-25.
3. Propst KL, Mima T, Choi KH, Dow SW, Schweizer HP. 2010. A *Burkholderia pseudomallei* delta*purM* mutant is avirulent in immunocompetent and immunodeficient animals: candidate strain for exclusion from select-agent lists. Infect Immun 78:3136-43.
4. Holloway BW. 1955. Genetic recombination in *Pseudomonas aeruginosa*. J Gen Microbiol 13:572-81.
5. Borlee BR, Goldman AD, Murakami K, Samudrala R, Wozniak DJ, Parsek MR. 2010. *Pseudomonas aeruginosa* uses a cyclic-di-GMP-regulated adhesin to reinforce the biofilm extracellular matrix. Mol Microbiol 75:827-42.
6. Choi KH, Mima T, Casart Y, Rholl D, Kumar A, Beacham IR, Schweizer HP. 2008. Genetic tools for select-agent-compliant manipulation of *Burkholderia pseudomallei*. Appl Environ Microbiol 74:1064-75.
7. Rholl DA, Papp-Wallace KM, Tomaras AP, Vasil ML, Bonomo RA, Schweizer HP. 2011. Molecular Investigations of PenA-mediated beta-lactam Resistance in *Burkholderia pseudomallei*. Front Microbiol 2:139.
8. Plumley BA, Martin KH, Borlee GI, Marlenee NL, Burtnick MN, Brett PJ, AuCoin DP, Bowen RA, Schweizer HP, Borlee BR. 2016. Thermoregulation of biofilm formation in *Burkholderia pseudomallei* is disrupted by mutation of a putative diguanylate cyclase. J Bacteriol doi:10.1128/JB.00780-16.
9. Biggins JB, Kang HS, Ternei MA, DeShazer D, Brady SF. 2014. The chemical arsenal of *Burkholderia pseudomallei* is essential for pathogenicity. J Am Chem Soc 136:9484-90.

Supplemental Table 3

Cluster	Full Locus	Product
1	BP1026B_I1731-I1736	Malleobactin
2	BP1026B_I1663-I1681	Unknown
3	BP1026B_I1157-I1176	Unknown
4	(not found in Bp 1026)	Not applicable
5	BP1026B_II2504-II2509	Unknown
6	BP1026B_II0147	Terphenyl
7	BP1026B_II0180-II0185	Isonitrile
8	BP1026B_II0328-II0340	Malleilactone
9	BP1026B_II0535-II0541	2-Alkyl-4-quinolone
10	BP1026B_II0641-II0648	Pyochelin
11	BP1026B_II1103-II1108	Unknown
12	BP1026B_II1232-II1254	Bactobolin
13	BP1026B_II1250-II1267	Unknown
14	BP1026B_II1345-II1353	Syrbactin
15	BP1026B_II1742-II1746	Malleipeptin
16	BP1026B_II1935-II1945	Unknown

Supplemental methods

Reverse transcriptase PCR

Total RNA was isolated from exponential phase cultures (OD_{600} 0.7 – 0.8) grown in 3 mL LB cultures at 37°C shaking at 250 rpm. Following double treatment with DNaseI (Ambion/Life Technologies), 500 ng RNA was reverse transcribed using random hexamers and the Transcriptor First Strand cDNA Synthesis Kit (Roche) following the protocol recommended by the manufacturer. To analyze co-transcription of Bp1026b_I2284 (*cdpA*) and Bp1026b_I2285, the cDNA was amplified using the following PCR primers: 5'- GACGCGATCAAGCTGACGGGC-3' and 5'-CGAACGCCATGTACATCGCCG-3'. This primer set generated a 719 bp amplicon spanning the intergenic region of *cdpA* and I2285. A high-fidelity KAPA polymerase was used with 5x GC buffer with the following cycle conditions: pre-incubation at 95°C for 30 s, followed by 25 cycles of denaturation at 98°C for 20 s, annealing at 62°C for 15 s, extension at 72°C for 15 s, and a final incubation at 72°C for 1 min. 50 µL reactions were split into duplicates and visualized on an agarose gel. Bp82 genomic DNA was included as a positive control and cDNA from Δ*cdpA*-I2285 mutant was included as a negative control.

Heterologous expression of *B. pseudomallei* c-di-GMP genes

Heterologous expression of *B. pseudomallei* c-di-GMP genes was done as described in the methods of this paper with the exception of triparental matings that were conducted with PAO1 and PAO1 Δ*wspF* as recipients.
